# Evaluating the Prognostic and Therapeutic Potentials of the Proteasome 26S Subunit, ATPase (*PSMC*) Family of Genes in Lung Adenocarcinoma: A Database Mining Approach

**DOI:** 10.3389/fgene.2022.935286

**Published:** 2022-07-22

**Authors:** Md. Asad Ullah, Nafisa Nawal Islam, Abu Tayab Moin, Su Hyun Park, Bonglee Kim

**Affiliations:** ^1^ Department of Biotechnology and Genetic Engineering, Faculty of Biological Sciences, Jahangirnagar University, Dhaka, Bangladesh; ^2^ Department of Genetic Engineering and Biotechnology, Faculty of Biological Sciences, University of Chittagong, Chattogram, Bangladesh; ^3^ Department of Pathology, College of Korean Medicine, Kyung Hee University, Seoul, Korea; ^4^ Korean Medicine-Based Drug Repositioning Cancer Research Center, College of Korean Medicine, Kyung Hee University, Seoul, Korea

**Keywords:** biomarker, diagnostic, lung cancer, *PSMC*s, therapeutic

## Abstract

This study explored the prognostic and therapeutic potentials of multiple Proteasome 26S Subunit, ATPase (*PSMC*) family of genes (*PSMC1-5*) in lung adenocarcinoma (LUAD) diagnosis and treatment. All the *PSMC*s were found to be differentially expressed (upregulated) at the mRNA and protein levels in LUAD tissues. The promoter and multiple coding regions of *PSMC*s were reported to be differentially and distinctly methylated, which may serve in the methylation-sensitive diagnosis of LUAD patients. Multiple somatic mutations (alteration frequency: 0.6–2%) were observed along the *PSMC* coding regions in LUAD tissues that could assist in the high-throughput screening of LUAD patients. A significant association between the *PSMC* overexpression and LUAD patients’ poor overall and relapse-free survival (*p* < 0.05; HR: >1.3) and individual cancer stages (*p* < 0.001) was discovered, which justifies *PSMC*s as the ideal targets for LUAD diagnosis. Multiple immune cells and modulators (i.e., CD274 and IDO1) were found to be associated with the expression levels of *PSMCs* in LUAD tissues that could aid in formulating *PSMC*-based diagnostic measures and therapeutic interventions for LUAD. Functional enrichment analysis of neighbor genes of *PSMC*s in LUAD tissues revealed different genes (i.e., *SLIRP, PSMA2,* and *NUDSF3*) previously known to be involved in oncogenic processes and metastasis are co-expressed with *PSMC*s, which could also be investigated further. Overall, this study recommends that *PSMCs* and their transcriptional and translational products are potential candidates for LUAD diagnostic and therapeutic measure discovery.

## Introduction

Lung adenocarcinoma (LUAD), which develops along the outer edge of the lungs within glandular cells in the small airways and falls under the umbrella of non-small cell lung cancer (NSCLC), is the most common type of histology, accounting for about 40% of all lung malignancies (Senosain and Massion, 2020; Zheng et al., 2020). Worldwide research on 185 countries suggests that about 11.4% (more than 2.2 million) new cases of lung cancer were diagnosed in 2020, with an almost 18% mortality rate (1.8 million deaths) (Sung et al., 2021). The low survival rate of patients with LUAD can be attributed to the lack of understanding of lung cancer biology, genomics, and host factors that drive the progression of preinvasive lesions, heterogeneity of disease, and patients’ outcomes. Although available diagnosis methods and treatment options have led to the overall decline in the mortality rate from this prevalent cancer, the 5-years survival rate remains below 20% (Hirsch et al., 2017; Myers and Wallen, 2022). Therefore, there is an increasing demand to secure an efficient diagnostic and therapeutic target for LUAD diagnosis and treatment that can significantly aid in the early-stage diagnosis, proper tracking of the patients throughout the cancer stages, and appropriate therapeutic interventions ultimately reducing the medical burden. Investigation of specific prognostic and therapeutic markers for disease stages or tumor types can help develop better screening strategies, improve patients’ prognoses, and assuage the financial burden of the disease (Hirsch et al., 2017; Oberndorfer and Müllauer, 2018; Devarakonda and Govindan, 2019; Zheng et al., 2020). Additionally, exploring molecular features, i.e., genetic variation, aberrant methylation, and immunophenotypes of specific targets can further increase the precision of cancer diagnosis and treatment.

The multiple Proteasome 26S Subunit, ATPase (*PSMC*) family of genes are reported to be involved in protein degradation, which plays a vital role in regulating the 26S proteasome (Kao et al., 2021). This family of genes is composed of six members, namely, *PSMC1, PSMC2, PSMC3, PSMC4, PSMC5,* and *PSMC6* (*PSMC1-6*) (Table 1). They partially constitute the formation of the 19S proteasome complex comprised of 19 essential subunits (Gu and Enenkel, 2014). This regulatory complex, in turn, catalyzes the unfolding and translocation of substrates into the 20S proteasome (Kao et al., 2021). Proteasomes control normal cellular function and maintain homeostasis by regulating the optimum degradation of different cellular proteins. However, the upregulated proteasome activity can greatly alter a broad range of crucial cellular processes i.e., DNA replication, transcription, cell cycle and apoptosis (Manasanch and Orlowski, 2017). Given its pivotal roles in the aberrant degradation of the mediators (i.e., activators and inhibitors) of cell cycle and apoptosis regulators upon overproduction in cancer cells, inhibition of the proteasome activity remains a promising target for anticancer therapy development (Sterz et al., 2008; Park et al., 2018).

**TABLE 1:** Genomic characteristics of all the subunits in the *PSMC* gene family.

Gene	Length of the nucleotide (bp)	Chromosomal location	Length of amino acids	Protein encoded	References
*PSMC1*	18,903	14q32.11	440	26S protease regulatory subunit 4 (26S proteasome AAA-ATPase subunit Rpt2)	(Tanahashi et al., 1998; National Center for Biotechnology Information (NCBI), 2022)
*PSMC2*	41,777	7q22.1-q22.3	433	26S protease regulatory subunit 7 (26S proteasome AAA-ATPase subunit Rpt1)	(Tanahashi et al., 1998; National Center for Biotechnology Information (NCBI), 2022; Shibuya et al., 1992)
*PSMC3*	7,705	11p11.2	439	26S protease regulatory subunit 6A (26S proteasome AAA-ATPase subunit Rpt5)	(Hoyle et al., 1997; Tanahashi et al., 1998)
*PSMC4*	10,615	19q13.11-q13.13	418	26S protease regulatory subunit 6B (26S proteasome AAA-ATPase subunit Rpt3)	(Tanahashi et al., 1998; National Center for Biotechnology Information (NCBI), 2022; Choi et al., 1996)
*PSMC5*	4,875	17q23.3	406	26S protease regulatory subunit 8 (26S proteasome AAA-ATPase subunit Rpt6)	(Tanahashi et al., 1998; National Center for Biotechnology Information (NCBI), 2022; Hoyle et al., 1997)
*PSMC6*	21,428	14q22.1	389	26S protease regulatory subunit S10B (26S proteasome AAA-ATPase subunit Rpt4)	(Tanahashi et al., 1998; National Center for Biotechnology Information (NCBI), 2022; Fujiwara et al., 1996)

As of now, multiple *PSMC* family genes have been studied in the context of different human diseases including carcinoma. For example, a previous study showed that *PSMC6* promotes osteoblast apoptosis and cancer cell proliferation by inhibiting the activation of the PI3K/AKT signaling pathway in an animal model of ovariectomy-induced osteoporosis (Zhang et al., 2020). *PSMC2* was found to be upregulated in osteosarcoma (Song et al., 2017), prostate cancer (Chen et al., 2021), pancreatic cancer (Qin et al., 2019), glioma (Zheng et al., 2022), oral squamous cell carcinoma (OSCC) (Wang et al., 2022), and hepatocellular carcinoma (HCC) (Ding et al., 2019; Li et al., 2019; Liu et al., 2021). Moreover, *PSMC2* was also reported to promote proliferation and inhibit apoptosis of glioma cells, and its knockdown halted the development and metastasis of prostate cancer (Chen et al., 2021) and progression of OSCC cells by promoting apoptosis *via* PI3K/Akt pathway and increasing the expression of pro-apoptotic proteins (Wang et al., 2022). *PSMC5* is involved in the ubiquitination-dependent degradation of Tln1 and angiogenesis by blocking the miR-214/PTEN/Akt pathway (Li et al., 2019). Knockdown of Proteasome 26S subunit ATPase 3 interacting protein (*PSMC3IP*) resulted in the suppression of xenograft proliferation and tumorigenesis in the HCC cells (Wang et al., 2022). In a recent study, researchers elucidated the crucial role of *PSMC* family members and their downstream-regulated genes in breast cancer progression (Kao et al., 2021). However, the collective potential of the *PSMC* family of genes as candidates to be novel prognostic biomarkers and therapeutic targets in LUAD remains to be unveiled. Out of the six *PSMC* subunits, a recent systematic study evaluated the differential expression levels and prognostic values of *PSMC6* as a high *PSMC6* expression was associated with poor prognosis of LUAD, indicating the potential of *PSMC6* as a promising therapeutic target for LUAD (Zhang et al., 2021). Though the study mentioned earlier focused on the prognostic power of *PSMC6* in LUAD, the molecular characterization, i.e., genetic alteration frequency, aberrant methylation, and immune phenotypes of the *PSMC* family genes in LUAD, which could further assist in diagnostic and therapeutic development, remains unstudied from a holistic perspective.

**FIGURE 1:**
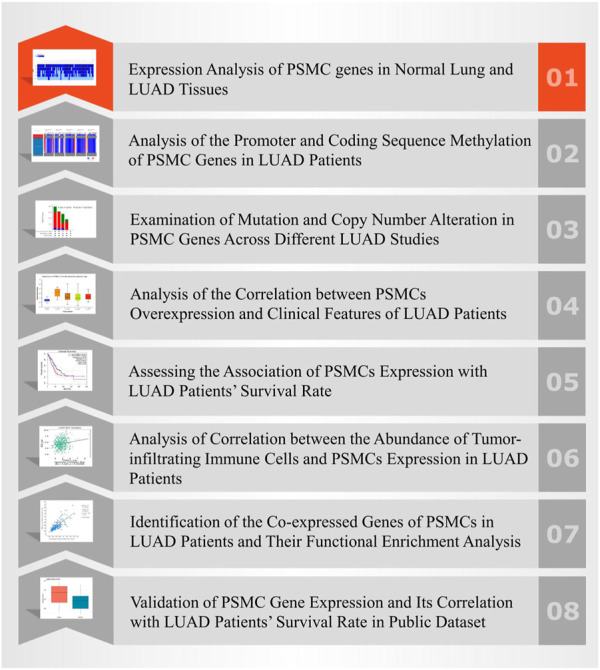
Strategies utilized in the database mining approach employed in the overall study.

This study evaluated the prognostic and therapeutic significance of the multiple Proteasome 26S Subunit, ATPase (*PSMC*) family of genes (*PSMC1-5*) in LUAD utilizing a web-based database mining approach. Since the prognostic value of *PSMC6* has been studied in the context of LUAD, this member of *PSMC* family was not considered in this study (Zhang et al., 2021). Using a bioinformatics approach, we attempted to determine the expression patterns, methylation patterns, mutations, and copy number alterations of the *PSMC* genes in LUAD tissues (Figure 1). Furthermore, we examined the correlation between *PSMC* overexpression and the clinical features and different survival rate of LUAD patients. We also assessed the association between *PSMC* expression and abundance of tumor-infiltrating immune cells and co-expressed genes of *PSMCs* and their functional enrichment in LUAD patients. Our study should contribute to understanding the predictive roles of *PSMCs* and their transcriptional and translational products in LUAD development, progression, and prognosis, which should help further research work and clinical development of *PSMC*-based diagnostics and therapeutics for LUAD.

## Materials and Methods

### Expression Analysis of *PSMC* genes in Normal Lung and LUAD Tissues

*PSMC* gene expression pattern at the mRNA level in normal lung and cancerous LUAD tissues was determined using the OncoDB server (http://oncodb.org/, accessed on: 7 April 2022). OncoDB is an online platform that allows users to explore the differential gene expression (DGE) pattern in normal and corresponding cancerous tissues from The Cancer Genome Atlas (TCGA) and Genotype-Tissue Expression (GTEx) databases (Su et al., 2007). The differential expression pattern of *PSMC*s was evaluated between the log2 TPM (transcript per million) normalized RNA sequencing data of LUAD and adjacent normal lung tissue samples in the OncoDB server. The result was then analyzed based on the log2 fold change (log2FC) and false discovery rate adjusted *p*-value cutoff of the DGE analysis. After that, the Expression Atlas (https://www.ebi.ac.uk/gxa/home, accessed on: 7 April 2022) web-based tool was utilized to discover the mRNA level expression pattern of the *PSMC*s in a total of 68 different types of LUAD cell lines (Papatheodorou et al., 2018). Finally, the Human Protein Atlas (HPA) (https://www.proteinatlas.org/, accessed on: 7 April 2022) server was used to determine the protein level expression of *PSMC*s in normal lung and LUAD tissues by analyzing the immunohistochemistry (IHC) images (at 200 µm length) of the LUAD samples and adjacent normal lung tissues (Pontén et al., 2008). The Pathology and Tissues modules of the HPA server were explored to optimize the differences in *PSMC* protein expression between normal and cancerous lung tissues.

### Analysis of the Promoter and Coding Sequence Methylation of *PSMC* Genes in LUAD Patients

The UALCAN server (http://ualcan.path.uab.edu/, accessed on: 7 April 2022) was utilized to examine the promoter methylation pattern of *PSMC* genes in LUAD tissues (Chandrashekar et al., 2017). TCGA database (integrated with UALCAN server) was selected as the basis set for the experiment. The analysis was carried out by performing a student’s t-test between the methylation data of the test (LUAD samples) and control (adjacent normal lung tissue samples) variables. Finally, the result was checked and validated based on a significant *p*-value cutoff of <0.05. Next, the methylation pattern of the DNA sequence of *PSMC* coding genes was studied using the UCSC Xena browser (https://xenabrowser.net/, accessed on: 7 April 2022) (Goldman et al., 2019). In this step, the integrated TCGA LUAD samples (*n* = 706) were again selected to observe the *PSMC* coding sequence methylation using the methylation 450k array data. The samples for which methylation data were not available were omitted during the analysis. Finally, the GSCA server was used to confirm the association between *PSMC* methylation and gene expression in LUAD tissues (http://bioinfo.life.hust.edu.cn/GSCA/#/, accessed on: 7 April 2022) (Liu et al., 2018). The impact of *PSMC* methylation on the survival rate of LUAD patients was also evaluated from the GSCA tool.

### Examination of Mutation and Copy Number Alteration in *PSMC* Genes Across Different LUAD Studies

The cBioPortal server (https://www.cbioportal.org/, accessed on: 7 April 2022) was accessed to analyze the mutation and copy number alteration (CNA) in *PSMC* genes across a wide number LUAD study samples (Gao et al., 2013). The data deposited by MSKCC, Broad, OncoSG, TDP, CPTAC, and others including more than 2,598 patients’ samples over nine studies were searched for *PSMC* mutation and CNA analysis in LUAD patients. The OncoPrint summary of the overall mutations of the selected *PSMC*s in different LUAD studies was inspected. Next, the bar diagram representing the type of genetic alterations in *PSMC* coding genes was also analyzed. The relation between the overall survival (OS) of LUAD patients and *PSMC* gene alteration was evaluated from this server. The parameter values were kept at defaults during the analysis in cBioPortal server. Finally, the correlation between the CNAs present in *PSMC*s their mRNA level expression in LUAD tissues was discovered in the form of a bubble plot using the mutation module in GSCA (accessed on: 7 April 2022) server.

### Analysis of the Correlation Between the *PSMC* Overexpression and Clinical Features of LUAD Patients

The association between the *PSMC* gene overexpression and LUAD patients’ clinical features and demographic status, i.e., age, individual cancer stages, and nodal metastasis status, was evaluated from the UALCAN server (accessed on: 7 April 2022). UALCAN is a comprehensive, user-friendly online tool that enables users to access omics data in cancer biomarker discovery and target validation. The TCGA LUAD samples were selected for the association analysis with our genes of interest in this study. The analysis result was considered significant based on the *p*-value cutoff of <0.05 found in the student’s t-test, and the expression profile was retrieved as box plots with transcript per million (TPM) reads unit.

### Assessing the Association of the *PSMC* Expression with LUAD Patients’ Survival Rate

The association between OS of LUAD patients and *PSMC* expression was established using the GEPIA 2 server (http://gepia2.cancer-pku.cn/, accessed on: 7 April 2022) (Tang et al., 2019). GEPIA 2 involves 9,736 tumors and 8,587 normal samples from GTEx and TCGA projects of RNA sequencing data, and this tool facilitates different transcriptional analyses, i.e., the analysis of correlation and differential expression across different normal and tumor tissues. Finally, the relation between the *PSMC* expression and LUAD patients’ relapse-free survival (RFS) was also determined from the GEPIA 2 (accessed on: 29 April 2022) server. The result of the experiment was then analyzed based on the *p*-value and hazard ratio (HR) of LUAD patients in relation to the differential level of *PSMC* expression represented in the Kaplan-Meier (KM) plot of survival analysis. The parameter values were kept at default during the analysis in GEPIA 2 server.

### Analysis of Correlation Between the Abundance of Tumor-Infiltrating Immune Cells and the *PSMC* Expression in LUAD Patients

The association between abundance of immune cells and *PSMC* expression in LUAD patients was determined utilizing the immune module of the GSCA database (accessed on: 7 April 2022). GSCA is a highly inclusive database that helps analyze different genomic association features and cancer patients’ clinical outcomes across different forms of cancer. Moreover, it also aids in the analysis of the correlation between different gene expressions, gene mutations, and the expression level of 24 different types of immune cells in different cancer patients. Every selected *PSMC* gene was queried against the abundance of immune cells like B Cells, CD8^+^ T cells, CD4^+^ T cells, macrophages, neutrophils, natural killer (NK) cells in LUAD microenvironment. Finally, the association between the *PSMC* expression and the abundance of different immunomodulators in LUAD patients was determined from the TISIDB server (http://cis.hku.hk/TISIDB/, accessed on: 7 April 2022) (Ru et al., 2019). The result of immune cell and modulator’s infiltration level was analyzed based on *p*-value and correlation coefficient.

### Identification of the Co-Expressed Genes of *PSMC*s in LUAD Patients and Their Functional Enrichment Analysis

The co-expressed genes of *PSMC*s were identified using the TCGA LUAD database (Firehose, Legacy) from the cBioPortal server (accessed on: 7 April 2022). After that, the top 300 positively co-expressed genes of each *PSMC* were selected based on *p*-value and correlation coefficient, which were then used to identify the overlapping neighbor genes utilizing the InteractiVenn online tool (http://www.interactivenn.net/, accessed on: 7 April 2022) (Heberle et al., 2015). The overlapping neighbor genes of *PSMC*s in LUAD tissues were then used in gene ontology terms, i.e., biological processes (BP), molecular function (MF), cellular component (CC), and Kyoto Encyclopedia of Genes and Genomes (KEGG) pathway analysis from the Enrichr server (https://maayanlab.cloud/Enrichr/, accessed on: 7 April 2022) (Kuleshov et al., 2016). The result of the functional enrichment analysis was then visualized and retrieved in the form bubble plot using the ImageGP online and publicly available tool (http://www.ehbio.com/ImageGP/, 7 April 2022) (Chen et al., 2022).

### Validation of the *PSMC* Gene Expression and its Correlation with LUAD Patients’ Survival Rate in the Public Dataset

In this step, we evaluated the pattern of *PSMC* mRNA expression in two independent microarray datasets, i.e., GSE1037 and GSE116959 from National Center for Biotechnology Information-Gene Expression Omnibus database (Barrett et al., 2012). GSE1037 contains total mRNA expression profiles of 105 lung cancer and adjacent normal lung tissue samples out of which 12 and 19 samples correspond to LUAD and adjacent normal tissues, respectively, which were utilized in our analysis (Jones et al., 2004). On the other hand, GSE116959 contains mRNA expression profiles of 57 LUAD and 11 adjacent normal lung tissue samples (Moreno Leon et al., 2019). Data normalization, log2 transformation, and expression value were calculated on the selected datasets using the BioConductor package in R studio (Gentleman et al., 2004; Allaire, 2012). The expression pattern was then visualized in the form of boxplot using ggplot2 package (Wickham et al., 2016). Moreover, we also examined the correlation between *PSMC1*-5 expression and LUAD patients' OS in GSE31210 microarray dataset that contains the clinical and mRNA expression profile of 226 LUAD patients (Okayama et al., 2012). A log-rank t test was applied between the higher and lower *PSMC* expressing LUAD patients using the survival and survminer packages in R studio and the result was retrieved in the form of KM plot (Kassambara et al., 2017).

## Results

### mRNA and Protein Level Differential Expression of *PSMC* Genes in Normal Lung and LUAD Tissues

**FIGURE 2:**
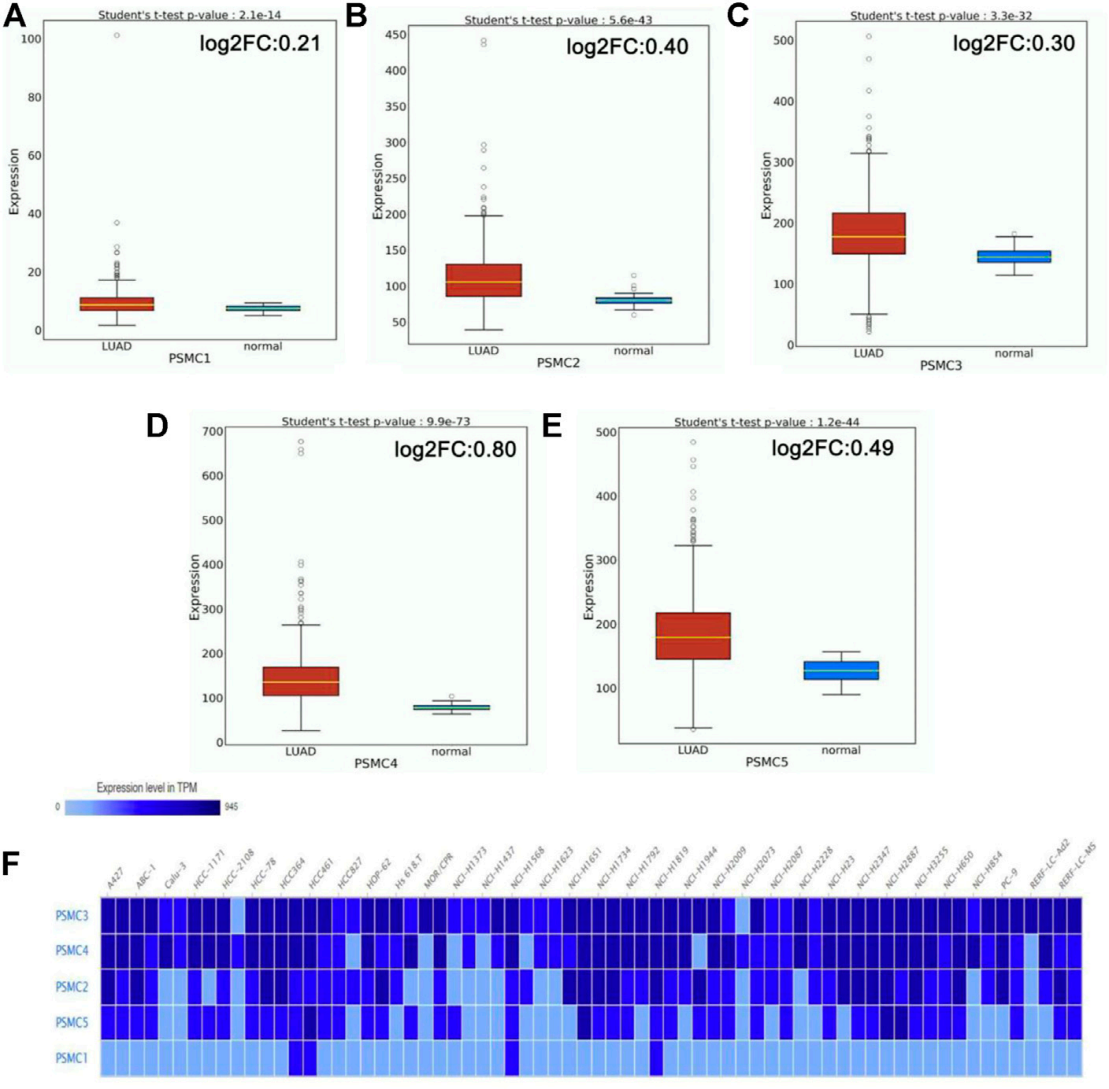
mRNA level differential expression patterns of *PSMC1* **(A)**, *PSMC2* **(B)**, *PSMC3* **(C)**, *PSMC4* **(D)**, and *PSMC5* **(E)** in normal lung and LUAD tissues observed from the OncoDB server. The expression values are presented in the TPM unit (log2 transformed). The red colored box represents LUAD samples, and the green colored box represents normal samples. The expression of the *PSMC*s in different LUAD cell lines **(F)**. The color gradient represents the expression value of *PSMC*s in TPM units in different cell lines, i.e., low intensity corresponds to a lower TPM and high intensity corresponds to a higher TPM, while the TPM value escalates with an increasing gradient from low to high. FC: fold change.

**FIGURE 3:**
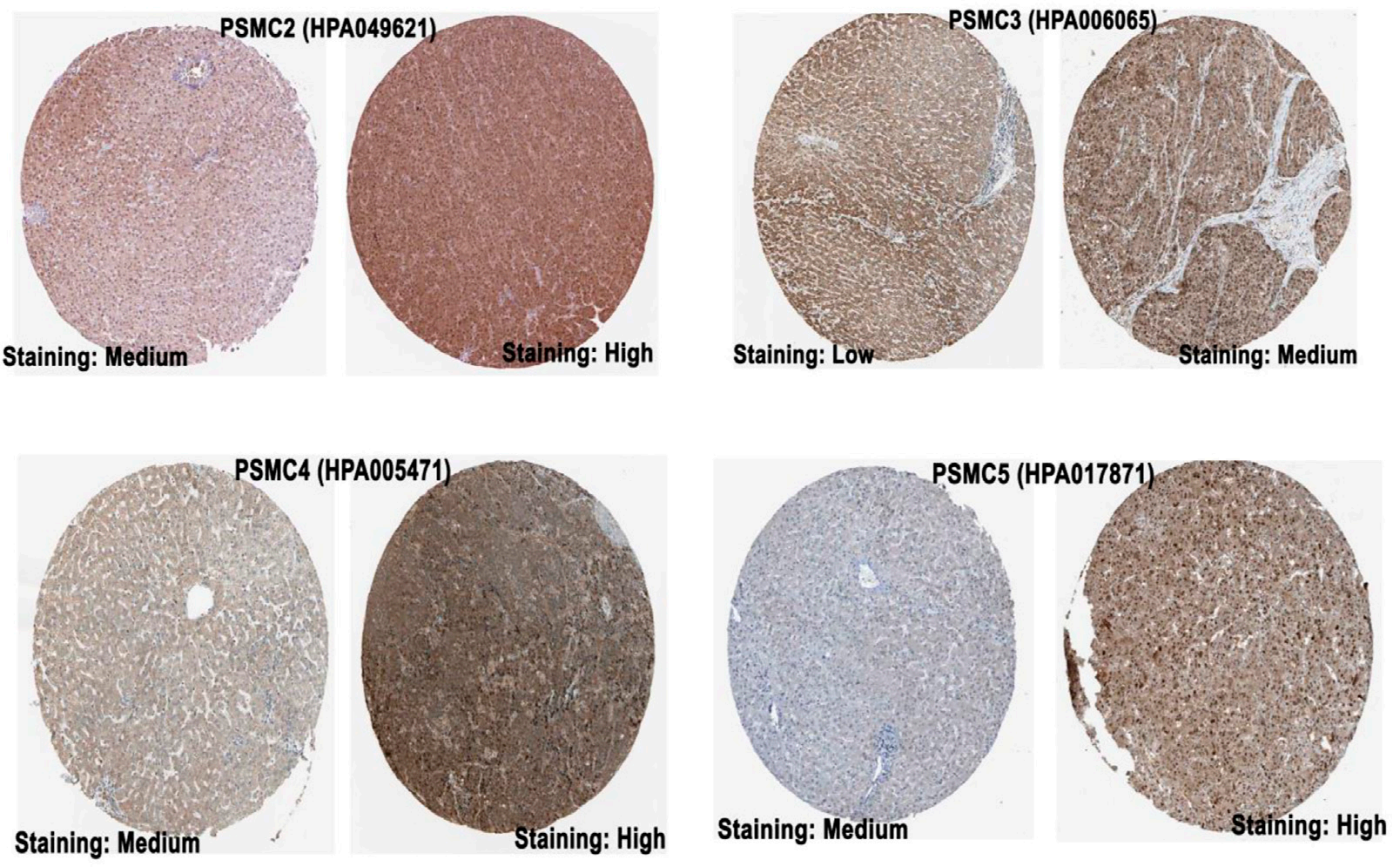
IHC images (visualized at 200 µm) delineating the protein level expression of *PSMC*s in normal lung (left) and LUAD tissue (right) from the HPA server. The name of the corresponding antibody used for IHC staining has been indicated inside the parentheses in addition to the gene name. The representative image for *PSMC1* was not found (Source: The Human Protein Atlas; https://www.proteinatlas.org/).

The mRNA level expression of *PSMC*s in normal lung and LUAD tissues was analyzed from the OncoDB server. All the *PSMC* genes showed higher expression levels in LUAD tissues than in normal lung tissues (Figure 2). Moreover, *PSMC4* (log2FC: 0.80) showed the highest difference of expression between the test and control group followed by *PSMC5* (log2FC: 0.49), *PSMC2* (log2FC: 0.40), *PSMC3* (log2FC: 0.30) and *PSMC1* (log2FC: 0.21). Thereafter, the expression pattern of our genes of interest was observed across 68 different LUAD cell lines and PSMC3 was discovered to be overexpressed inmost of the selected cell lines followed by *PSMC4* and *PSMC2* (Figure 2F). On the contrary, as in par with the previous result, *PSMC1* showed the least overexpression in all the LUAD cell lines. Overall, all the selected *PSMC*s showed higher expression levels in HCC461 and NCI-H1819 cell lines. Thereafter, the protein level expression pattern of the *PSMC*s in LUAD and their corresponding normal tissues was analyzed from the HPA server. *PSMC2* showed medium staining against the administered antibody (HPA049621) in normal lung tissues, whereas a stronger staining was recorded in the LUAD tissues (Figure 3). *PSMC3* demonstrated a low staining pattern in the normal lung tissues and medium staining in the LUAD tissues. Moreover, both *PSMC4* and *PSMC5* exhibited medium staining in the normal lung tissues, whereas a high level of staining was observed in the LUAD tissues.

### Promoter and Coding Sequence Methylation Status of *PSMC* Coding Genes in LUAD Tissues

**FIGURE 4:**
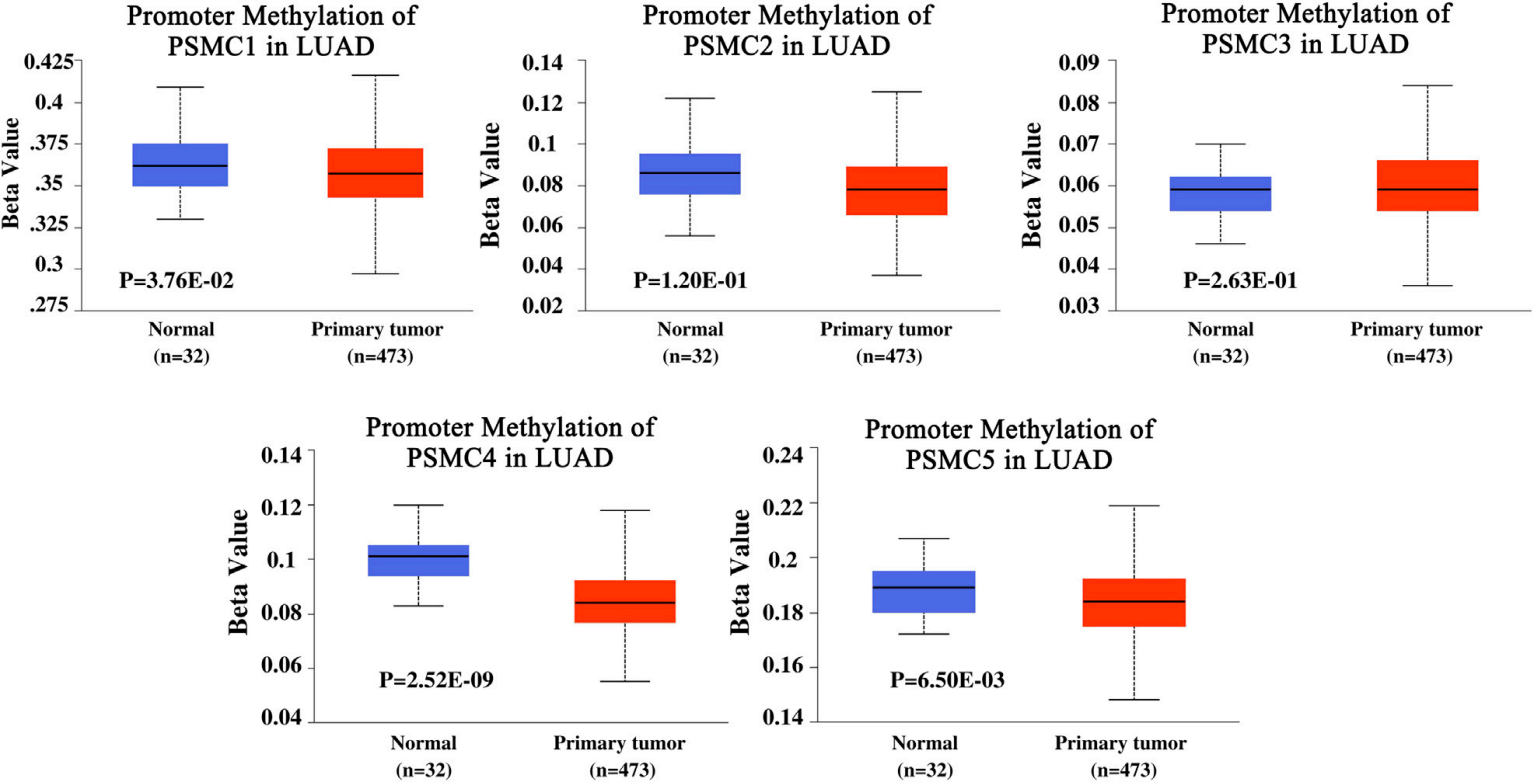
Promoter methylation pattern of *PSMC* genes in LUAD and normal lung tissues. Significant and distinct differential methylation patterns of different *PSMC*s in LUAD tissues was observed compared to the normal lung tissues. Normal: samples were collected from normal tissues adjacent to the cancerous tissues of LUAD patients within TCGA cohorts without any demographic and clinical stratification (Source: The Cancer Genome Atlas). Beta value cut-offs in the range of 0.7 to 0.5 indicate hypermethylation; 0.3–0.25 indicates hypomethylation.

The promoter methylation pattern of the *PSMC* genes in LUAD and normal lung tissues was examined from the UALCAN server. *PSMC1* gene coding promoter in LUAD tissues was found to be less methylated than in the normal lung tissues (*p* = 3.76e-02) (Figure 4). Although the *PSMC2* and *PSMC3* promoters were observed to be less methylated in LUAD tissues, the association was not significant (*p* > 0.05). Additionally, *PSMC4* (*p* = 2.52e-09) and *PSMC5* (*p* = 6.50e-03) promoters were also found to be less methylated in LUAD tissues compared to the normal lung tissues. The coding sequence methylation analysis of the *PSMC*s in LUAD tissues from the UCSC Xena browser revealed that the selected *PSMC*s might have distinct coding sequence methylation patterns. For example, *PSMC2*, *PSMC3,* and *PSMC4* signified that their coding regions might have the mostly methylated regions at the 3’ end of the sequence as indicated by the elevated beta value in red-colored regions (Supplementary Figure S1). On the contrary, *PSMC1* showed a completely different pattern of methylation in which most of the CpG islands might cover the 5’ end and a slight upstream region from the 3’ end of the coding sequence. In the case of the *PSMC5* methylation pattern, the red landscapes at the 5’ end indicated that the initial region of the coding sequence might be hypermethylated. Finally, the effect of methylation in *PSMC* genes on their mRNA expression level specific to LUAD tissues was determined from the GSCA server. Unsurprisingly, methylation was negatively correlated with the *PSMC1*, *PSMC2*, *PSMC4,* and *PSMC5* mRNA expression in LUAD tissues (Supplementary Figure S2). Additionally, *PSMC5* hypomethylation was found to be associated with poor OS (*p* = 0.043) and progression-free survival (PFS) (*p* = 0.017) in LUAD patients (Supplementary Figure S3).

### Frequency of *PSMC* Mutation and Copy Number Alterations in LUAD Tissues

**FIGURE 5:**
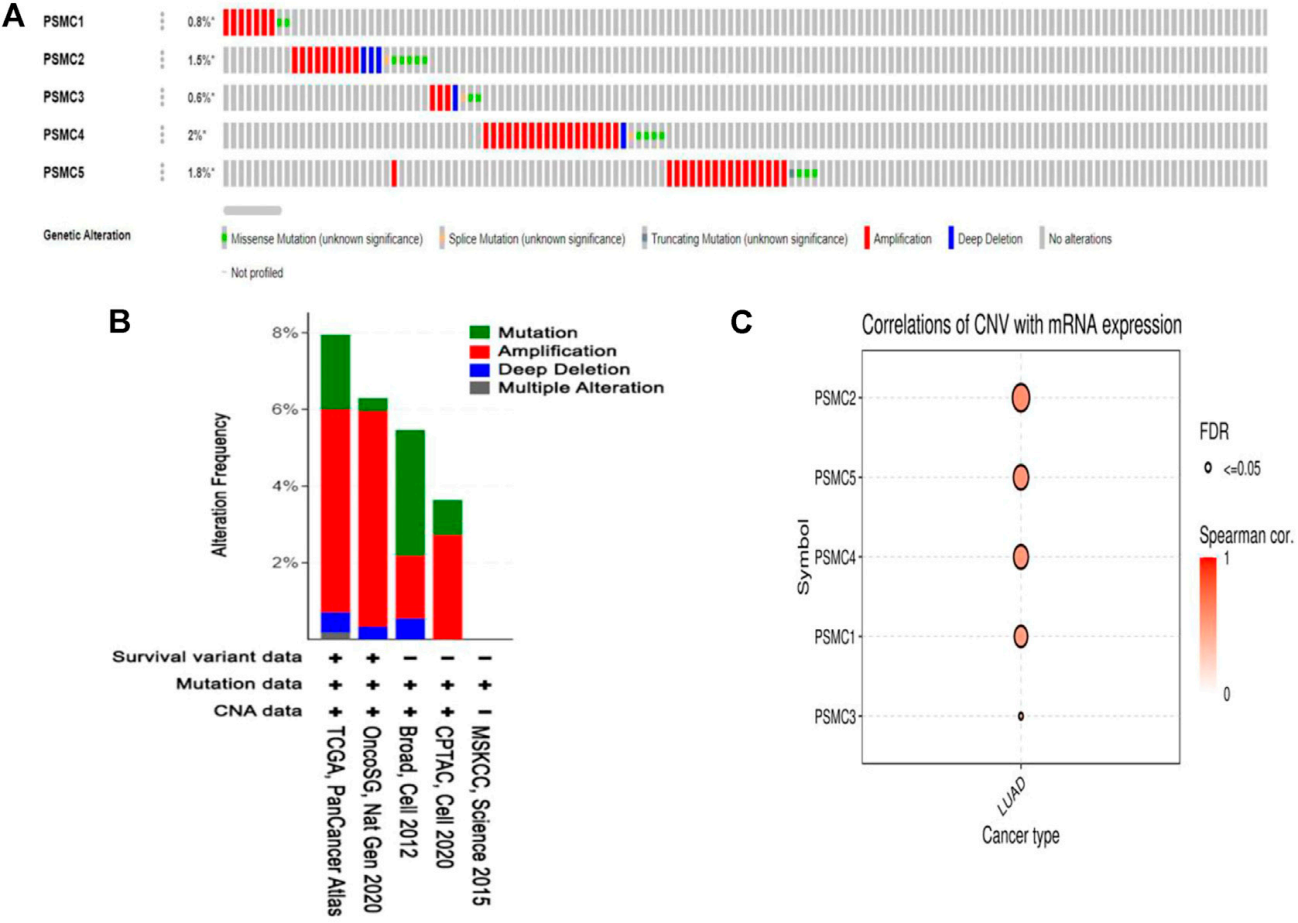
Mutation analysis report on *PSMC* genes in LUAD patients presented in OncoPrint diagram **(A)**. The colored region of the column represents different alterations, and the alternation frequency is a fraction of the colored columns over the total columns. Amplification was found to be the most prevalent form of genetic alteration in all *PSMC* genes across different LUAD studies. The distribution of mutation and CNA in *PSMC* genes across different LUAD studies is oriented in bar diagram **(B)**. The *y*-axis represents the percentage of total samples mutated in each LUAD study correspondingly presented in the *x*-axis. Bubble plot representing the positive correlation between the CNAs present in *PSMC* genes and their mRNA level expression in LUAD tissues **(C)**.

The mutation and copy number alteration frequency of *PSMC* genes across different LUAD studies were evaluated from the cBioPortal online tool. *PSMC1*, *PSMC2*, *PSMC3*, *PSMC4*, and *PSMC5* showed an alteration frequency of 0.8, 1.5, 0.6, 2, and 1.8%, respectively, across different LUAD studies (Figure 5A). The analysis reported the presence of different detrimental genetic alterations i.e., amplification, deep deletion, and splice sites across the *PSMC* coding regions in LUAD samples. Moreover, a number of missense mutations was also recorded in the *PSMC* coding regions carrying the potential to interfere with the protein functions. The copy number alteration (CNA) frequency analysis revealed the shreds of evidence of a large number of amplification events across the selected LUAD studies responsible for the genetic alterations in *PSMC* genes in LUAD patients (Figure 5B). Thereafter, the association between *PSMC* CNA in LUAD tissues and their expression patterns was established from the GSCA server. All the *PSMC*s showed a significant and positive correlation between the number of CNA events and their expression levels (Figure 5C). Finally, the effect of the mutations and CNAs present in *PSMC* genes on LUAD patients’ OS was also determined from this server. It was observed that *PSMC* mutations are significantly and negatively correlated to the LUAD patients’ OS (*p* = 1.19e-04) (Supplementary Figure S4). Altogether, *PSMC*s altered LUAD patients had a poor OS (median survival ∼49 months) compared to the unaltered patients (median survival ∼66 months).

### Association Between the *PSMC* Overexpression and LUAD Patients’ Clinical Features

**FIGURE 6:**
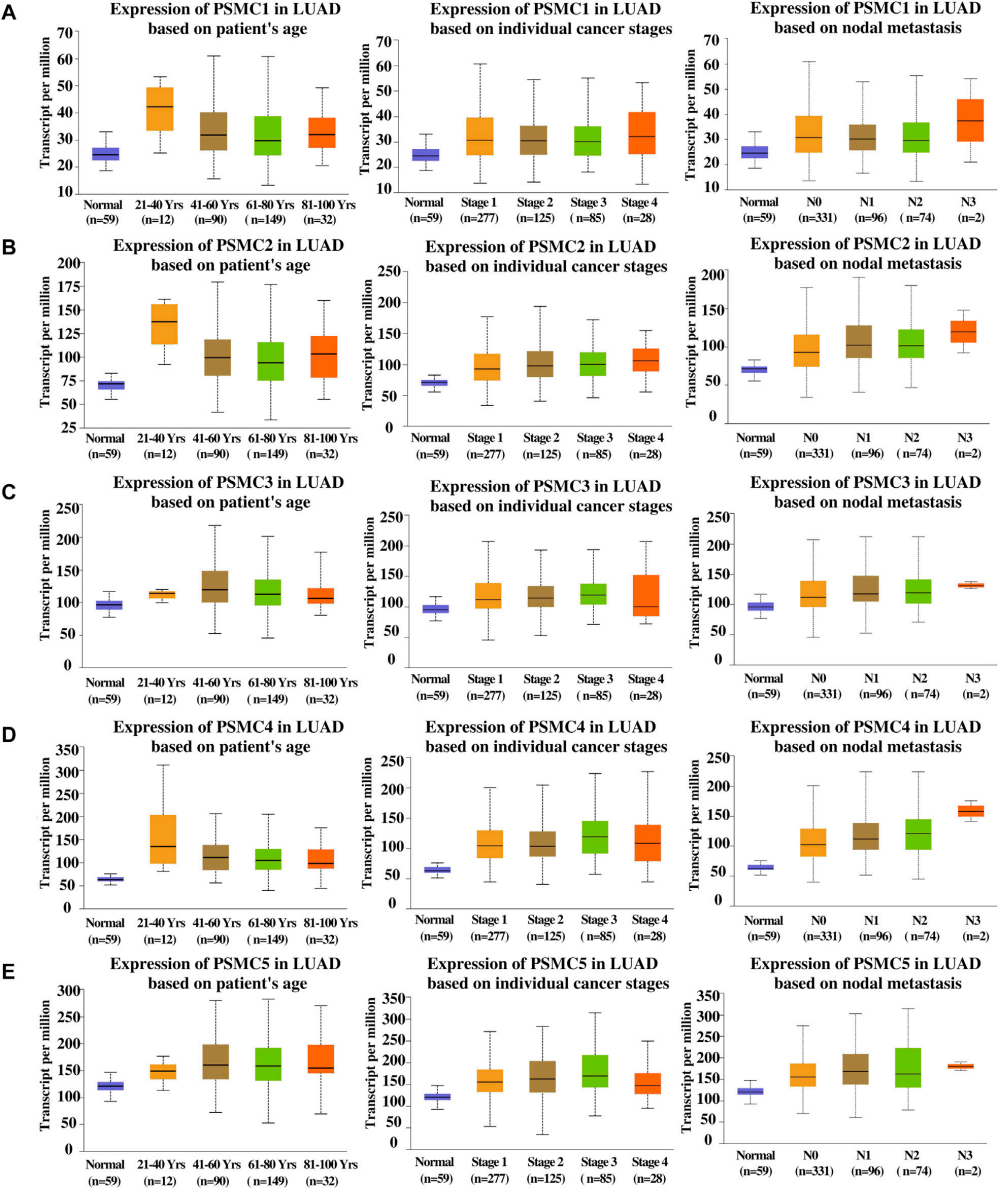
Pattern of *PSMC1* **(A)**, *PSMC2* **(B)**, *PSMC3* **(C)**, *PSMC4* **(D)**, and *PSMC5* **(E)** overexpressions in relation to LUAD patients’ age, individual cancer stages, and nodal metastasis status represented in box plots. Several significant associations between *PSMC*s expression levels and LUAD patients’ clinical features were observed. Normal: samples collected from normal tissues adjacent to the cancerous tissues of LUAD patients within TCGA cohorts without any demographic and clinical stratification (Source: The Cancer Genome Atlas). Please correspond to Supplementary Table S1 for a detailed observation of the clinical parameters.

The association between the *PSMC* overexpression and LUAD patients’ clinical characteristics was determined from the UALCAN server. Although a noticeable rise in the *PSMC* expression in the 21–40 years age group compared to the normal was found, *PSMC1*, *PSMC3,* and *PSMC4* genes did not show significant association as observed from student’s t-test performed between normal and cancerous samples (*p* > 0.05) (Supplementary Table S1) (Figure 6). Moreover, *PSMC1*-4 showed a marginal reduction in the expression among other age groups except for 21–40 years in LUAD tissues though the level remained above the normal lung tissue expression level whereas *PSMC5* showed a significant increase in expression in accordance with advancing age groups (*p* < 0.05) (Figure 6). On the contrary, in terms of cancer stages, *PSMC1* showed the highest expression level in stage 1 and stage 4 whereas the intermediate stages showed a downward trend in expression in LUAD tissues although the expression level still remained above that in normal lung tissues (*p* < 0.001) (Supplementary Table S1) (Figure 6). In the case of other *PSMC*s, all the genes showed a marginal increment in their expression levels in accordance with aggressive cancer stages with a slight decline in stage 4 in LUAD tissues (*p* < 0.05). As in par with the *PSMC1* expression level in comparison with cancer stages, its expression level was also found at the highest threshold in N0 and N3 of the nodal metastasis status group in LUAD patients. However, the association between the expression level of *PSMC1*, *PSMC2,* and *PSMC4* and N3 lymph node metastatic groups was not discovered to be significant (*p* > 0.05) (Supplementary Table S1) (Figure 6). In contrast, other *PSMC*s showed significant overexpression in accordance with the advancing metastasis stage in LUAD patients (*p* < 0.04).

### Relation Between the *PSMC* Expression and LUAD Patients’ Survival Rate

**FIGURE 7:**
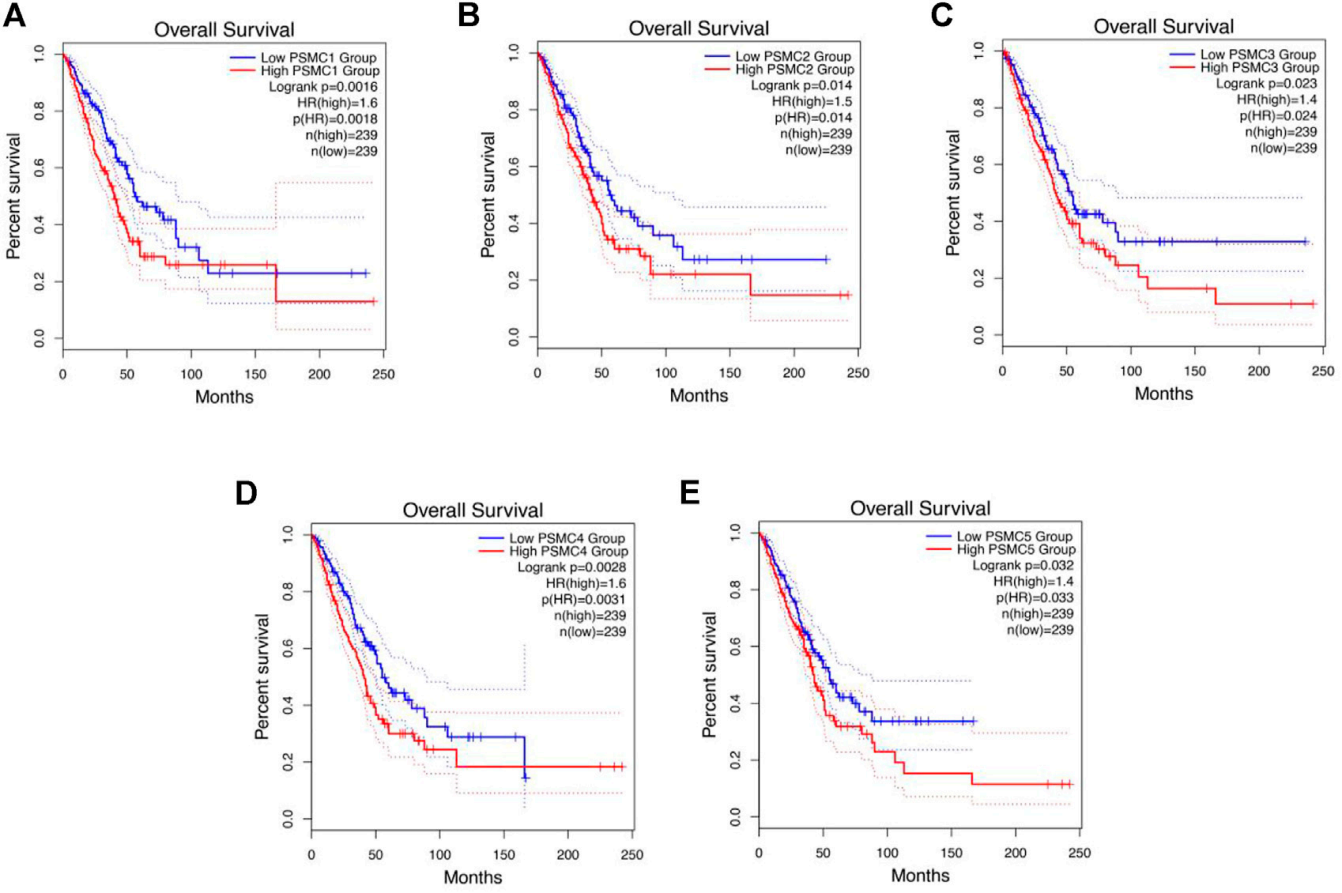
Kaplan–Meier plot representation of *PSMC1* **(A)**, *PSMC2* **(B)**, *PSMC3* **(C)**, *PSMC4* **(D)**, and *PSMC5* **(E)** expressions and their relation with the OS of LUAD patients. The red color plot represents the high *PSMC*-expressing LUAD patients, and the blue color plot represents the low *PSMC*-expressing group. The vertical tick mark within the plot indicates an event (death). A significant negative association was observed between *PSMC*s expression and LUAD patients’ OS (*p* < 0.05, HR: <1.4).

The relation between *PSMC*s expression and LUAD patients’ OS and RFS was established using the GEPIA 2 server. The report of the survival analysis was retrieved in the form of Kaplan–Meier plot. The analysis revealed that *PSMC1* overexpression is negatively correlated with the OS of LUAD patients [*p* = 0.0016; Hazard Ratio (HR): 1.6] (Figure 7A). Moreover, the *PSMC1* overexpression was also responsible for the poor RFS of LUAD patients (*p* = 0.011; HR: 1.5) (Supplementary Figure S5A). Similarly, *PSMC2* overexpression was discovered to be associated with the worsening OS (*p* = 0.014; HR: 1.5) in LUAD patients (Figure 7B). Remarkably, the *PSMC2* overexpression was found to be responsible for poor RFS in LUAD patients as observed by an HR of 1.2 (*p* = 0.008) (Supplementary Figure S5B). In the case of *PSMC3* expression, its overexpression was significantly and negatively correlated to the OS (*p* = 0.023; HR: 1.4) (Figure 7C). Though the association between *PSMC3* and LUAD patients’ RFS was not found to be significant, a noticeably lower *p*-value of 0.077 and an HR of 1.3 suggested that the overexpression of *PSMC3* might be responsible for poor RFS (Supplementary Figure S5C). A significant association was also observed between *PSMC4* overexpression and the poor OS of LUAD patients from the report of the survival analysis (*p* = 0.003; HR: 1.6) (Figure 7D). Again, the expression of *PSMC4* was found to be accounted for the worse RFS in LUAD patients also (*p* = 0.0079; HR: 1.2) (Supplementary Figure S5D). Finally, the *PSMC5* expression was also discovered to be negatively associated with the OS (*p* = 0.03; HR: 1.4) and RFS (HR: 1.3) of LUAD patients, though the association with RFS was not significant (*p* = 0.089) (Figure 7E) (Supplementary Figure S5E).

### Association Between the *PSMC* Expression and the Abundance of Tumor-Infiltrating Immune Cells and Immune Modulators in LUAD Patients

**FIGURE 8:**
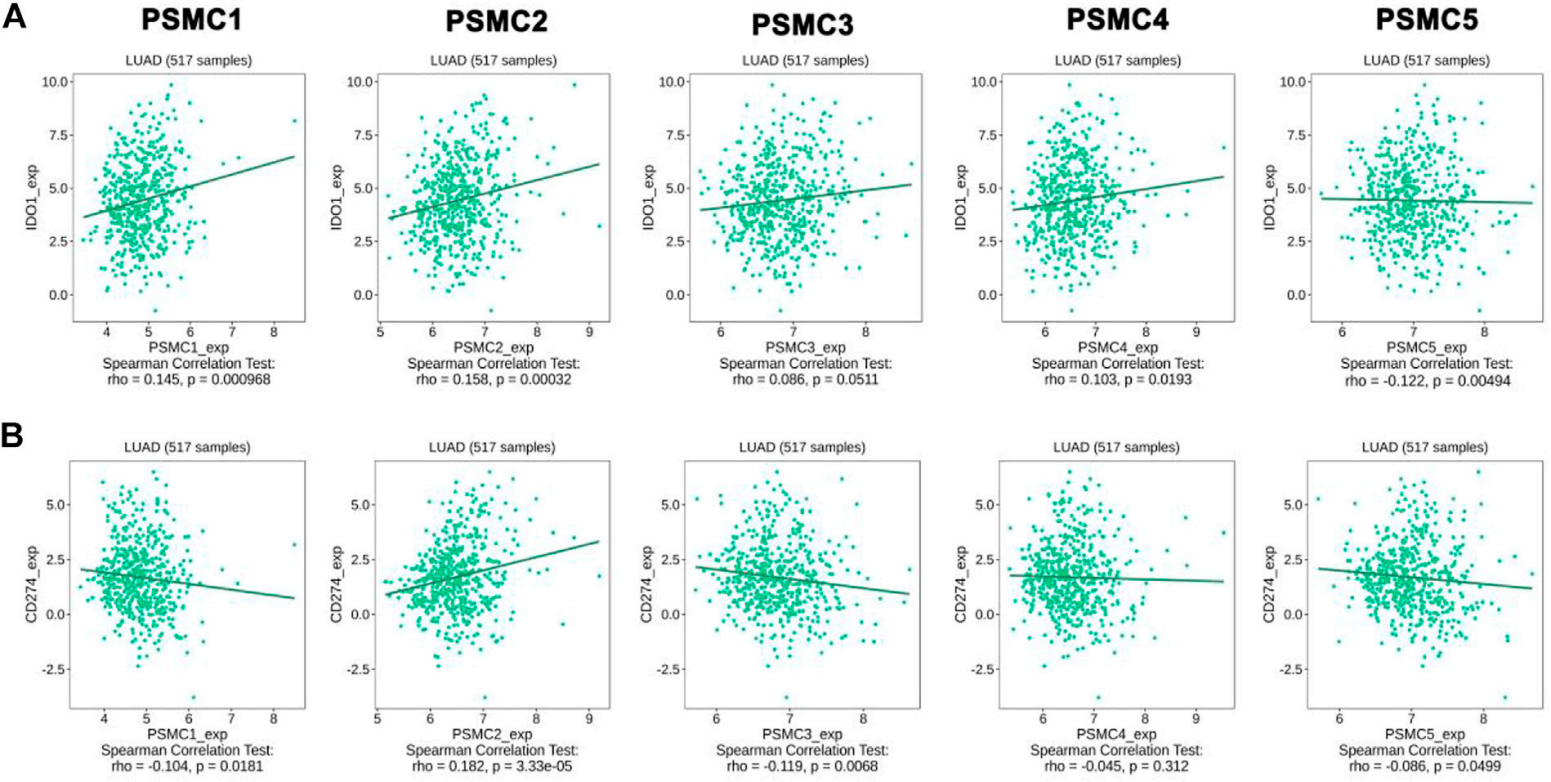
Association between the *PSMC* expression and the infiltration levels of IDO1 **(A)** and CD274 **(B)** in LUAD tissues. Significant positive and negative correlations were observed in between *PSMC* expression levels and immunomodulator expression levels.

The association between the *PSMC* expression and the abundance of different immune cells in LUAD tissues was evaluated from the GSCA server. A significant positive correlation between *PSMC1* expression and B cell (Cor: 0.09; *p* = 0.016) and CD8^+^ T cell (Cor: 0.13; *p* = 0.009) was observed in LUAD tissues. However, a negative correlation was observed between CD4^+^ T cell (Cor: −0.27; *p* = 3.29e-11) and *PSMC1* expression (Supplementary Table S2). *PSMC2* expression showed significant association with the abundance level of B cell (Cor: 0.16; *p* = 6.97e-05), CD8^+^ T cell (Cor: 0.09; *p* = 0.02) and dendritic cell (DC) (Cor: 0.21; *p* = 1.13e-07). On the contrary, *PSMC2* expression showed significant negative correlation with CD4^+^ T cell (Cor: -0.36; *p* = 2.34e-19), Natural Killer (NK) cell (Cor: -0.14; *p* = 0.0006), and Neutrophil (Cor: -0.12; *p* = 0.02) abundance level in LUAD and other surrounding tissues (Supplementary Table S2). In case of *PSMC3* expression, it showed significant positive association with B cell (Cor: 0.14; *p* = 0.004), CD8^+^ T cell (Cor: 0.15; *p* = 0.002), and Dendritic Cell (DC) (Cor: 0.12; *p* = 0.003). However, *PSMC3* exhibited negative association with CD4^+^ T cell (Cor: −0.28; *p* = 7.01E-12), NK cell (Cor: −0.13; *p* = 0.001) and Neutrophil (Cor: −0.08; *p* = 0.04) infiltration levels in LUAD patients (Supplementary Table S2). *PSMC4* and *PSMC5* showed a positive association with B cell, CD8^+^ T cells, monocytes and DC (Cor: > 0.09, *p* < 0.05) abundance levels in LUAD microenvironment. A significant negative association between the CD4^+^ T cell, NK cell, Macrophage and Neutrophil production level and *PSMC4* and *PSMC5* expression levels was observed (*p* < 0.05) (Supplementary Table S2). Thereafter, the association between the *PSMC*s overexpression and different immunoinhibitor infiltration levels in LUAD and adjacent tissues in the microenvironment was established from the TISIDB server. Later, the immunomodulators that showed significant association, i.e., CD274 and IDO1 with the *PSMC* expression, were inspected. *PSMC1*, *PSMC2*, *PSMC4*, and *PSMC5* showed positive correlation with IDO1 expression levels in LUAD tissues (*p* < 0.05) (Figure 8A). Moreover, the CD274 abundance levels in LUAD tissues showed a negative association with *PSMC1*, *PSMC3*, *PSMC4*, and *PSMC5* expression levels in LUAD tissues (*p* < 0.05) (Figure 8B). Only *PSMC2* showed a positive association with CD274 infiltration levels.

### Co-Expressed Genes of *PSMC*s in LUAD Tissues and Their Functional Enrichment Analysis

**FIGURE 9:**
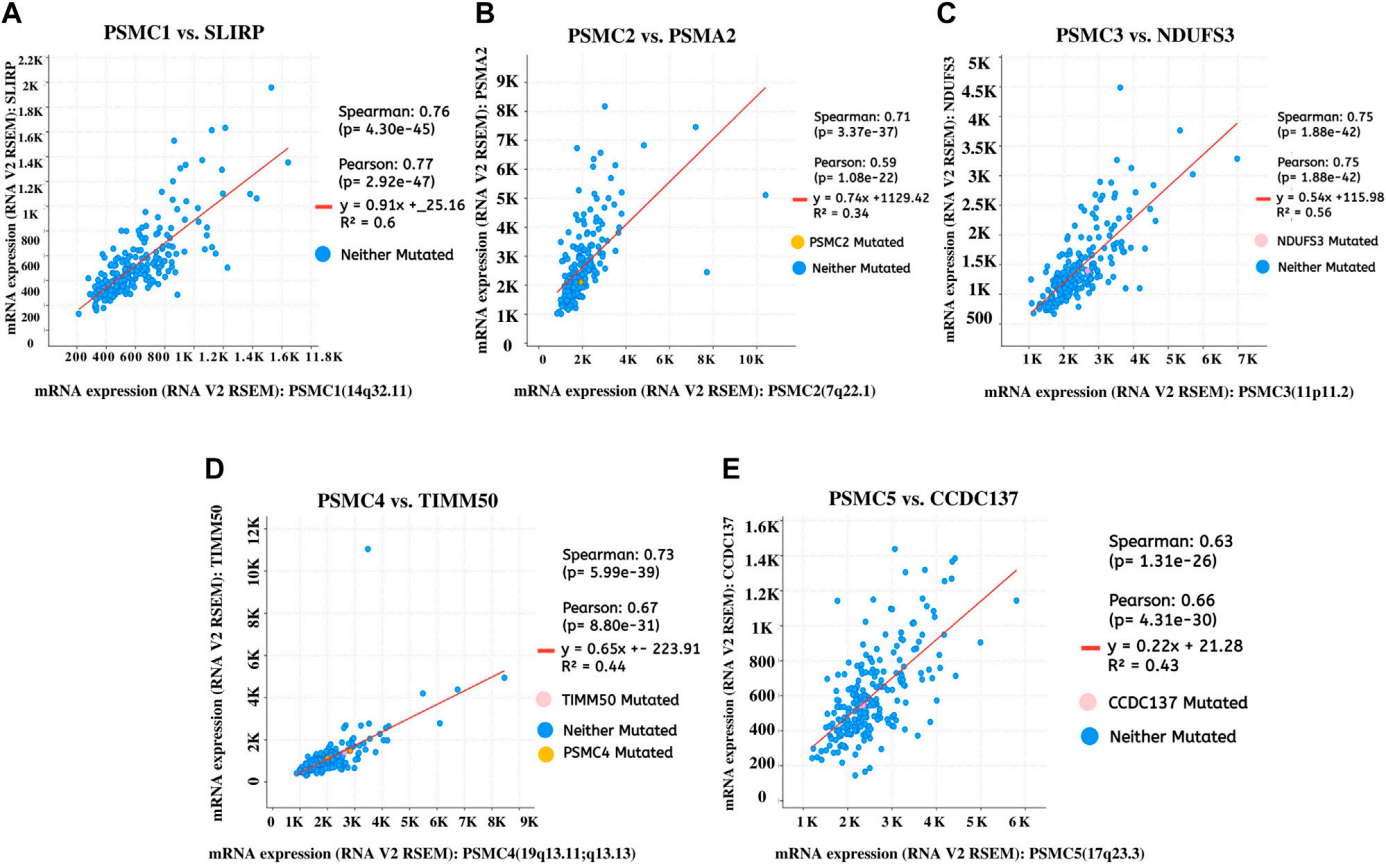
Top positively co-expressed genes of *PSMC1* **(A)**, *PSMC2* **(B)**, *PSMC3* **(C)**, *PSMC4* **(D)**, and *PSMC5* **(E)** in LUAD tissues obtained from the TCGA LUAD study (Firehose Legacy) through the cBioPortal server. The expression values were compared in the form RNA-seq V2 RSEM normalized scores of the sequencing reads. RSEM: RNA-seq by expectation maximization.

**FIGURE 10:**
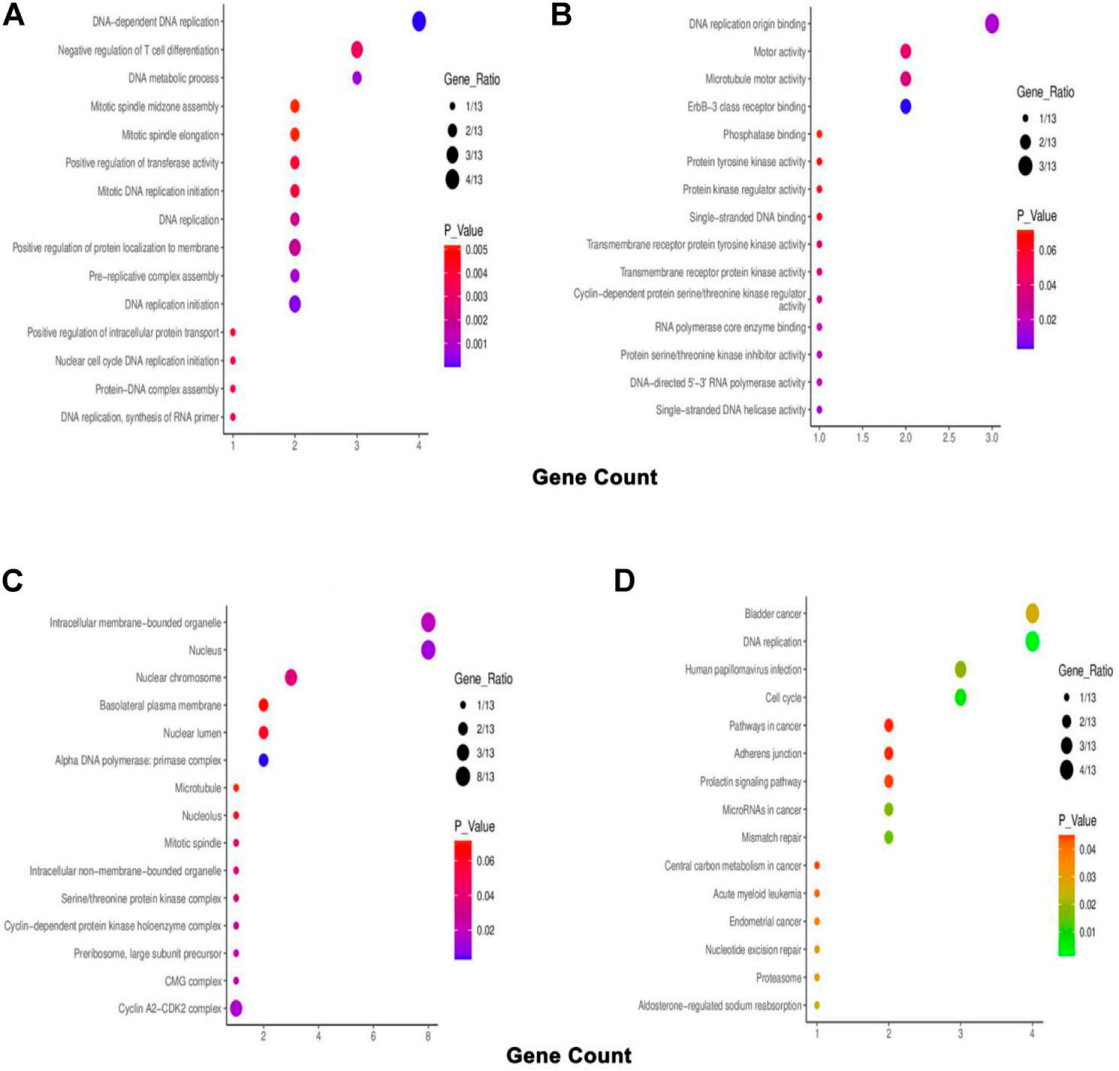
Bubble plots representing the enriched gene ontology terms of the overlapping 13 neighbor genes of *PSMC*s in LUAD tissues: **(A)** biological processes, **(B)** molecular function, **(C)** cellular component, and **(D)** KEGG pathway.

The top co-expressed gene of each *PSMC* in LUAD samples was identified from the cBioPortal server. *PSMC1* was discovered to be highly co-expressed with the SRA stem-loop interacting RNA-binding protein (*SLIRP*) coding gene in LUAD tissues (Cor: 0.76, *p* = 4.30e-45) (Figure 9A). Proteasome 20S subunit alpha 2 (*PSMA2*) gene showed the highest co-expression association with the *PSMC2* gene (Cor: 0.76, *p* = 4.30e-45) (Figure 9B). Moreover, the *PSMC3* gene was found to be most highly co-expressed with the NADH: Ubiquinone Oxidoreductase Core Subunit S3 (*NDUFS3*) gene in LUAD tissue samples (Cor: 0.75; *p* = 1.88e-42) (Figure 9C). Lastly, *PSMC4* and *PSMC5* genes were observed to have the highest level of co-expression with Translocase of Inner Mitochondrial Membrane 50 (*TIMM50*) (Cor: 0.75; *p* = 5.99e-39) and Coiled-coil Domain Containing 137 (*CCDC137*) genes (Cor: 0.63, *p* = 1.31e-26), respectively (Figures 9D,E). The analysis report also suggested the presence of mutated copies of both the *PSMC* genes and co-expressed genes within the samples except for *PSMC1*. Thereafter, the overlapping neighbor genes from the top 300 positively co-expressed genes of each *PSMC* family member in LUAD tissues were identified using the Venn diagram (Supplementary Figure S6). The analysis revealed 13 genes, i.e*., PSMB3, MRTO4, RFC2, TACO1, PRIM1, MCM3, KIF23, CCNA2, ERBB2, IRF1, PDCD45, SFN,* and *TOX3,* which are overlapped among the top 300 positively co-expressed genes of *PSMC*s. Afterward, the overlapping neighbor genes were investigated to understand their differential expression pattern in LUAD tissues. All the genes (except for *PDCD45*) showed significant overexpression in LUAD tissues compared to the normal lung tissues and only *IRF1* showed under-expression (Supplementary Figure S7). Finally, the overlapping genes were used in the functional enrichment analysis delineating different gene ontology terms, i.e., biological processes, molecular functions and cellular components, and the KEGG pathway. The biological process analysis revealed that the highest ratio of the genes is involved in DNA replication, negative regulation of T-cell differentiation, DNA metabolic processes, and mitotic spindle assembly (Figure 10A). The major molecular functions of the queried genes were DNA replication origin binding, phosphatase binding, motor activity, and microtubule motor activity (Figure 10B). The overlapping genes were predominantly operating in intracellular membrane-bound organelles, nucleus, and basolateral plasma membrane as observed from the cellular component analysis (Figure 10C). The KEGG pathway analysis on the overlapping neighbor genes of *PSMC*s in LUAD tissues reported that most of the genes are involved in pathways associated with bladder cancer, DNA replication, cell cycle, human papillomavirus infection, and so forth (Figure 10D).

### Validation of the Differential Expression and Clinical Relevance of *PSMC* Genes in LUAD Tissues from the Public Dataset

**FIGURE 11:**
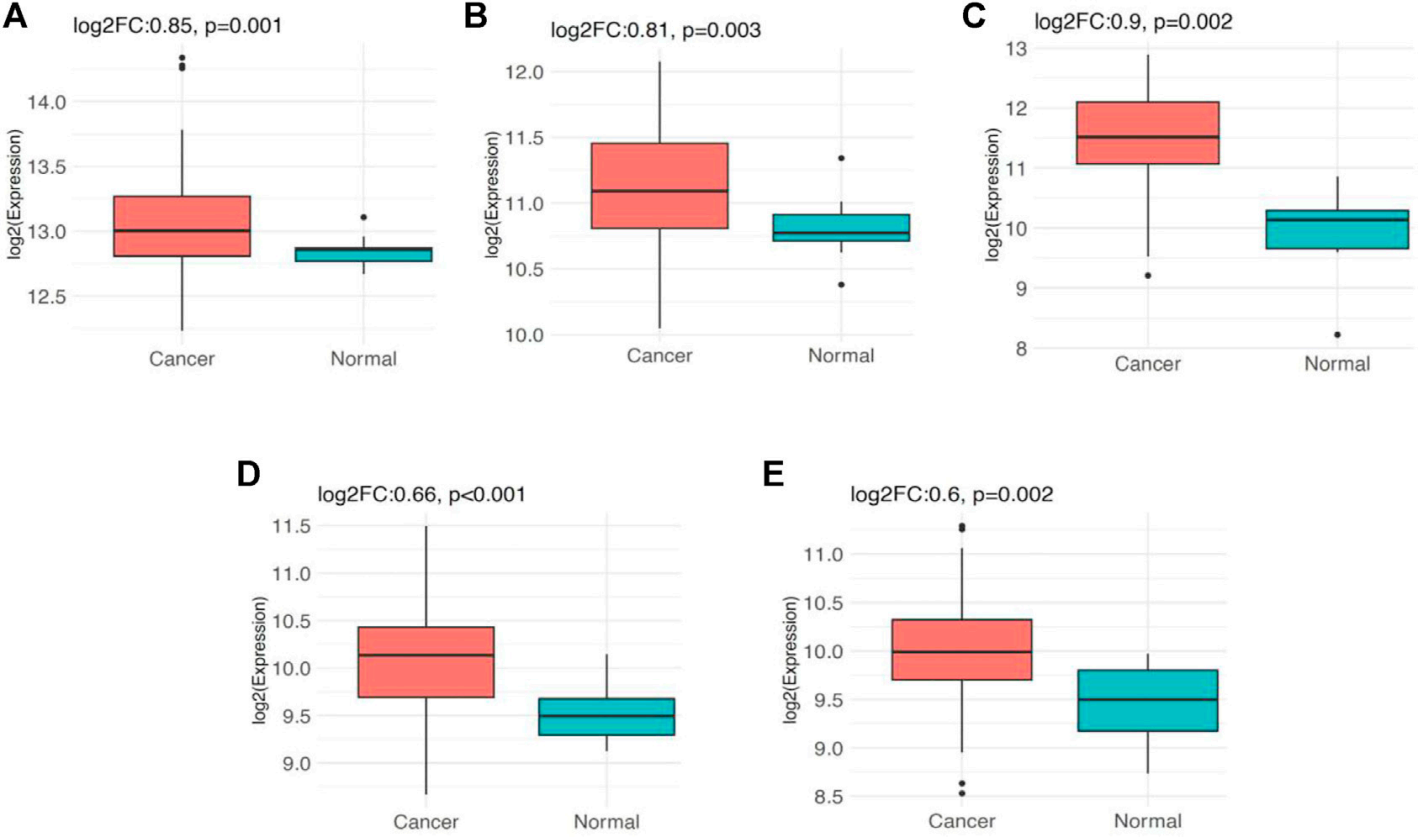
Expression pattern of the *PSMC* genes in LUAD tissues (*n* = 57) and adjacent normal lung tissues (*n* = 11) obtained from the GSE116959 dataset: **(A)** *PSMC1*, **(B)** *PSMC2*, **(C)** *PSMC3*, **(D)** *PSMC4*, and **(E)** *PSMC5*. All the *PSMC*s were found to be significantly overexpressed in LUAD tissues compared to the adjacent normal lung tissues (log2FC: >0.59, *p* < 0.05)

The analysis of *PSMC* gene expression in GSE116959 dataset revealed that *PSMC1* gene is significantly overexpressed (log2FC: 0.85; *p* = 0.001) in LUAD tissues (*n* = 57) compared to the adjacent normal lung tissues (*n* = 11). Similarly, *PSMC2* (log2FC: 0.81; *p* = 0.003), *PSMC3* (log2FC: 0.9; *p* = 0.002), *PSMC4* (log2FC: 0.66; *p* < 0.001) and *PSMC5* (log2FC: 0.6; *p* = 0.002) also showed overexpression in LUAD tissues compared to the normal tissues (Figure 11). Additionally, the expression analysis of *PSMC* genes in GSE1037 dataset also reported that all the *PSMC*s (*PSMC1*-5) are significantly overexpressed (log2FC: >0.66; *p* < 0.05) in LUAD tissues (*n* = 12) than in normal lung tissues (*n* = 19) (Supplementary Figure S8). Thereafter, we examined the association between the *PSMC* expression and LUAD patients’ OS from GSE31210 (*n* = 226) dataset by performing a log-rank t-test between the higher and lower *PSMC-*expressing patients. Though the overexpression of *PSMC1* was found to be negatively associated with the OS of LUAD patients, the correlation was not discovered to be significant. On the contrary, *PSMC2*-5 overexpression was reported to be significantly and negatively associated with the OS of LUAD patients (*p* < 0.05) (Supplementary Figure S9).

## Discussion

This study explored the prognostic values of the *PSMC* family of gene expression in LUAD, taking advantage of the database mining approach. Initially, the differential expression pattern of all the selected *PSMCs* in LUAD and its corresponding adjacent normal lung tissues was evaluated. Given that cancer development is a multistep process controlled by various biological processes, differential gene expression analysis allows the understanding of the possible involvements of particular genes in the oncogenic development of a healthy cell (Bashyam, 2002; Liang and Pardee, 2003; Kim et al., 2015). This study found that all the *PSMC*s are highly expressed in LUAD tissues compared to the normal lung tissues both at the mRNA and protein level, suggesting their possible functions in LUAD development and progression (Figure 2 and Figure 3). Although the expression pattern of the selected genes (*PSMC1*-5) in LUAD tissues remains unstudied to date, previously *PSMC6* was found to be overexpressed at both mRNA and protein levels in LUAD tissues than in adjacent normal lung tissues (Zhang et al., 2021). Moreover, all the genes of our interest also showed higher expression levels in different LUAD cell lines.

DNA methylation is one of the epigenetic drivers in cancer development and progression. Usually, promoter methylation regulates the gene activation and silencing and aberrant methylation is commonly associated with the up or downregulation of different genes in cancer cells (Liang and Pardee, 2003; Kulis and Esteller, 2010). Moreover, coding sequence methylation can also control the gene activity by altering the nucleosome orientation inside the chromatin structure (Ehrlich, 2002; Luczak and Jagodziński, 2006). Additionally, the reduced methylation of different genes can propel the tumorigenesis of healthy cells inside the human body by escalating the activity of different oncogenes and thus differential methylation remains a promising target for epigenetic clinical decisions in cancer treatment (Issa, 2007). In this experiment, the promoter regions of all the PSMCs were found to be differentially methylated (less methylated) in LUAD tissues compared to the normal lung tissues (Figure 4). Thus, the overexpression of the *PSMCs* in LUAD tissues may be attributed to the less methylated regions in *PSMC* coding promoters. In a recent study, *PSMC5* methylation has been linked to being negatively associated with colorectal cancer exacerbation (He et al., 2021). Furthermore, the evidence on the association between *PSMC5* hypomethylation and LUAD patients’ poor OS and RFS reveals the potential of PSMCs in epigenetic-based therapeutic discovery for LUAD treatment (Supplementary Figure S3). Moreover, the differentially methylated circulating genes from samples like urine or blood of cancer patients can serve as a diagnostic marker for early-stage detection of lung cancer (Hong and Kim, 2021). In this experiment, several different regions of *PSMC* coding sequences have been found to have distinct methylation patterns across LUAD samples which along with the differential level of promoter methylation may aid in the noninvasive diagnosis of LUAD patients (Figure 4).

Somatic driver mutations are the major etiological factors in LUAD development. Hence, the optimum understanding of the genetic alterations in relevant genes and their relations to patients’ survival is paramount (Pao and Girard, 2011). Unsurprisingly, CNA contributes more to the oncogenic development and subsequent growth of healthy cells than other nonsynonymous mutations like point mutations (Zhao et al., 2004; Vikberg et al., 2017). In our study, all the *PSMCs* were predicted to have multiple somatic alteration events, including amplification, deep deletion, and splice which could promote the LUAD exacerbation by alternating the dosage of the translation products of the *PSMCs* inside the cells. In support of such assumptions, *PSMC* mutations in this study were associated with the poor OS of LUAD patients (Figure 5). What’s more, the presence of multiple missense mutations as evidenced in *PSMC* coding regions in LUAD patients may also influence the LUAD development and progression by producing non-functional, dysfunctional, or entirely no protein (Figure 5). Although the roles of *PSMC* gene mutations in cancer remain unstudied, multiple mutations in other proteasome family genes, i.e., *PSMB5*, *PSMB6*, and *PSMB7*, are associated with the myeloma cell survival (Shi et al., 2020). Apart from this, the prevalence of CNA events in different protein-coding genes can aid in the high-throughput diagnosis of lung cancer patients. For example, previously, different CNAs, including both loss and gain events in human chromosomes 3 and 6 have been successful in the high-throughput diagnosis of lung cancer within 44 months with an accuracy of 97% (Bowcock, 2014). Thus, this study’s observed alterations in *PSMC* genes could also be investigated in formulating *PSMC*-based diagnostic measures for the early stage and accurate screening of LUAD patients.

Furthermore, all the *PSMC* genes were found to be overexpressed at an earlier age in the LUAD patients. A significant increase in the expression levels of the genes was observed across different cancer stages and with advancing lymph node metastasis status (Figure 6). The expression of *PSMC* genes was negatively associated with OS and RFS of LUAD patients (Figure 7). These pieces of evidence suggest that *PSMC*-based diagnostic measures may serve as a practical diagnosis method that could allow the early-stage diagnosis and tracking of LUAD patients throughout the clinical courses.

Tumor-infiltrating immune cells play a crucial role in inhibiting cancer cell growth and different immune cells have been shown to improve the prognosis of lung cancer patients (Wang et al., 2019). Apart from this, the abundance of immune cells in cancer patients can also aid in tracking the patient’s status throughout the disease state and formulating immunotherapy for use during the clinical course (Bremnes et al., 2016). For example, previous studies have shown that the abundance of CD4^+^, CD8^+^ T cells, and neutrophils are prognostic factors in lung cancer (Woo et al., 2001; Eruslanov et al., 2014; Djenidi et al., 2015). In this study, a significant association between *PSMCs* expression and different immune cells infiltration including the ones mentioned earlier was observed in LUAD patients that might assist in propagating dual diagnosis along with the *PSMC*-based diagnosis method (Figure 7). On the other hand, the mutated form of the *PSMC* expression can alter the immune reactivity in the cancer microenvironment and worsen the prognosis of LUAD patients. Additionally, multiple *PSMC* mRNA expression levels were found to be positively and negatively correlated with the infiltration levels of different immunomodulators like CD274 (commonly known as programmed death-ligand 1; PD-L1) and indoleamine 2,3-dioxygenase 1 (IDO1) enzyme (Figure 8). PD-L1 is the most frequently found cell surface receptor in NSCLC, and its overexpression predicts poor survival of lung cancer patients. Additionally, PD-L1 checkpoint inhibition and anti-PD-L1 antibodies are the most widely studied immunotherapy approaches in lung cancer, as well as, anti-PD-L1 antibodies are approved by the Food and Drug Administration for IHC-based diagnosis of lung cancer (Steven et al., 2016; Ancevski Hunter et al., 2018). Moreover, IDO1 is a promising anticancer target for different cancer treatments including lung cancer whose function can be regulated by small candidate molecules and the process provides immune blockade opportunities outside the immune checkpoint inhibition and adoptive immune cell transfer (Du et al., 2019). Therefore, IDO1 and CD274 could guide the *PSMC*-based diagnostic methods and therapeutic option discovery for LUAD.

The co-expression analysis revealed that *SLIRP* is the top and highly co-expressed gene of *PSMC1* which was shown to have prognostic roles in colorectal cancer (Salama et al., 2009). Among other selected top co-expressed genes, *PSMA2* was found to promote colorectal cancer cell proliferation and *NDUFS3* has been reported to be downregulated in the ovarian cancer cell and hypothesized to promote oncogenic development (Wang et al., 2013; Qi et al., 2021) (Figure 9). *TIMM50*, a gene found to be highly co-expressed with *PSMC4*, promotes tumorigenesis and acts as a prognostic indicator in NSCLC (Zhang et al., 2019). Moreover, in a study involving 129 colorectal cancer (CRC) patients, *TIMM50* was discovered as a key regulator and prognostic marker of CRC (Sun et al., 2020). A pan-cancer analysis recently reported that *CCDC137* plays a crucial role and acts as a prognostic marker in different forms of cancers (Guo et al., 2021). Given that the co-expressed genes are functionally related, all these shreds of evidence suggest that the *PSMC* family of genes might have an underlying mechanism in the oncogenic development of healthy lung cells.

Furthermore, all the positively co-expressed overlapping genes of *PSMCs* except *PDCD45* in LUAD tissues were also found to be primarily associated with the LUAD development (Supplementary Figure S7). Gene ontology term analysis on these genes suggested that most are involved in DNA replication, controlling the cell cycle, and operating in the nucleus. The KEGG pathway analysis indicated that the genes are predominantly involved in different cancer pathways, development of bladder cancer, oncogenic virus infection pathways, and so on. These findings again signify that the *PSMC* family genes may be associated with LUAD development and progression since the deregulation of the activity of *PSMCs*’ co-expressed genes in LUAD tissues can result in the initiation of oncogenic processes (Byler et al., 2014). As a result, the co-expressed genes of the *PSMCs* could also be investigated in LUAD therapeutic and diagnostic measures discovery. However, further laboratory investigations are required on such assumptions. Finally, the overexpression pattern of *PSMC*s in LUAD tissues was evaluated in two independent small-scale (*n* = ∼20–60) microarray datasets (i.e., GSE116959 and GSE1037) while the mainstream analysis involved large-scale (*n* = ∼500–700) RNA sequencing data from LUAD samples of TCGA and GTEx cohorts. All the selected genes of this study also showed significant overexpression in the LUAD samples from the independent microarray datasets (Figure 11) (Supplementary Figure S8). The survival analysis of LUAD patients in relation to *PSMC* expression in the public dataset (GSE31210) also supported our initial analysis that overexpression of most of the *PSMC*s (*PSMC2*-5) is significantly and negatively associated with the OS of LUAD patients (Supplementary Figure S9).

Overall, this study demonstrated the differential expression of *PSMCs* in LUAD patients at both mRNA and protein levels. There was a significant association between *PSMC*s overexpression and LUAD patients’ clinical manifestation. Moreover, *PSMCs* overexpression was correlated to the poor OS and RFS of LUAD patients. All these pieces of evidence suggest that the transcriptomic and proteomic differential expression patterns of *PSMC*s could assist in the *PSMC*-based LUAD diagnosis. Moreover, their upregulation pattern in LUAD tissues may also be responsible for an elevated level of proteasome activities ultimately leading to the LUAD development and growth by aberrantly degrading the regulators of the cell cycle and apoptosis (Sterz et al., 2008; Park et al., 2018). Hence, the abnormal expression pattern of *PSMC*s in LUAD patients, irrespective of their demographic and clinical conditions, suggests that *PSMC*s could be a potential target for LUAD treatment option discovery. Moreover, all the genes of our interest showed variation in the methylation pattern of promoters and coding sequences between normal lung and LUAD tissues. Several missense and truncating mutations were reported in the *PSMCs* coding regions. Additionally, *PSMC* expression was found to be associated with different immune cells and immune modulators in LUAD microenvironment. Thus, the findings on genetic and epigenetic alterations and immune phenotypes of *PSMC*s may aid in preparing and increasing the precision of *PSMC*-based diagnostic and therapeutic approaches against LUAD. More specifically, out of the five selected *PSMC*s, *PSMC4* showed the highest overexpression at the mRNA level (log2FC:0.8) and its overexpression was recorded in most of the LUAD cell lines. *PSMC4* was also found to have the least methylated promoters in LUAD tissues among all the other *PSMC*s. Genetic alteration frequency was also the highest number for *PSMC4* (2%) in LUAD tissues. Again, it also showed the highest HR (1.6) along with *PSMC1* against the OS of LUAD patients. These indicate that *PSMC4* might have the most prognostic power among the selected *PSMC*s in detecting the LUAD patients. However, such trajectories of our analysis require further investigation, and other *PSMC*s should also be investigated as they showed quite similar reports. Last, the functional enrichment analysis unveiled different co-expressed genes controlling biological processes during the cell cycle. Thus, the neighbor genes of *PSMCs* could also be investigated further while extending laboratory work on making *PSMC*-based diagnostic and therapeutic measures for LUAD patients. Overall, this study suggests that *PSMCs* and their transcriptional and translational products are efficient prognostic and therapeutic targets for LUAD diagnosis and treatment. The scientific findings of this study should aid in advancing further research on *PSMC*-based diagnostic and therapeutic development for LUAD and translating *PSMC*s into clinical practice.

Lastly, this study involved a large number of datasets to establish the prognostic and therapeutic potentials of *PSMC*1-5 in LUAD and most of the analysis was found to be significant. Later, the overexpression pattern and the association of these genes with LUAD patients’ survival rate were validated in a small-scale dataset and the inquiry was on par with our mainstream analysis. Moreover, this study provided multi-omics, i.e., genomic, transcriptomic, and proteomic overview of *PSMC* gene expression in LUAD prognosis. However, this study has some limitations, i.e., this study could not provide clearer insights into the molecular pathogenesis of *PSMC* genes in LUAD which requires further inspection. Alongside, though this study demonstrated the differential promoter methylation pattern of *PSMC*1-5 genes in LUAD, it could not testify whether the coding sequence of these genes is aberrantly methylated in LUAD tissues or not. Hereby, we warrant further laboratory research to extend the findings of this study which is currently underway by the authors.

## Data Availability

The original contributions presented in the study are included in the article/Supplementary Material; further inquiries can be directed to the corresponding author.
