# Supplementary material for: Evaluating the Prognostic and Therapeutic Potentials of the Proteasome 26S Subunit, ATPase (PSMC) Family of Genes in Lung Adenocarcinoma: A Database Mining Approach
Source: Front Genet. 2022 Jul 22;13:935286. doi: 10.3389/fgene.2022.935286 (PMC9353525; doi:10.3389/fgene.2022.935286)
Supplement: Supplementary file 1 [file DataSheet1.docx]

**Identifying Proteasome 26S Subunit, ATPase (*PSMC*) Family Genes as the Prognostic Indicators and Therapeutic Targets in Lung Adenocarcinoma**

Md. Asad Ullah^1^, Nafisa Nawal Islam^1^, Abu Tayab Moin^2^, Su Hyun Park^3^ and Bonglee Kim^3, 4^*^,^

^1^Department of Biotechnology and Genetic Engineering, Faculty of Biological Sciences, Jahangirnagar University, Dhaka, Bangladesh. [ullah1194@gmail.com](mailto:ullah1194@gmail.com) (M.A.U.); [nafisanawal@juniv.edu](mailto:nafisanawal@juniv.edu) (N.N.I.)

^2^Department of Genetic Engineering and Biotechnology, Faculty of Biological Sciences, University of Chittagong, Chattogram, Bangladesh. [tayabmoin786@gmail.com](mailto:tayabmoin786@gmail.com) (A.T.M.)

^3^Department of Pathology, College of Korean Medicine, Kyung Hee University, 1-5 Hoegidong, Dongdaemun-gu, Seoul, 02447, Republic of Korea. [kmdsoo@khu.ac.kr](mailto:kmdsoo@khu.ac.kr) (S.H.P.); [bongleekim@khu.ac.kr](mailto:bongleekim@khu.ac.kr) (B.K.)

^4^Korean Medicine-Based Drug Repositioning Cancer Research Center, College of Korean Medicine, Kyung Hee University, Hoegi-dong Dongdaemun-gu, Seoul, 02447, Republic of Korea;

*Corresponding Author

**Supplementary Information**

**
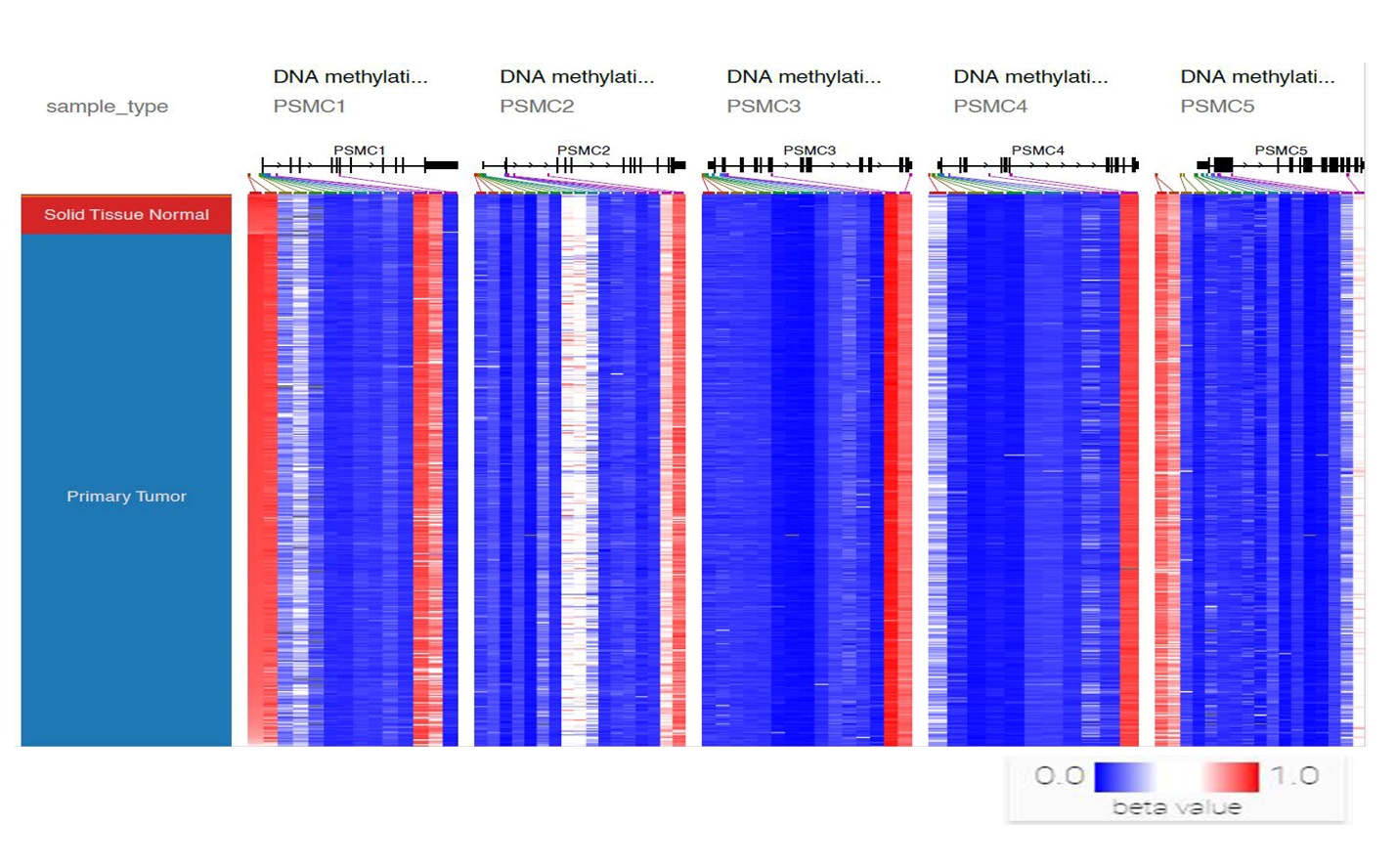
**

**Supplementary Figure S1:** The promoter coding sequence methylation pattern of *PSMC* genes in LUAD lung tissues in comparison with sample type. Significant and distinct differential methylation pattern of the *PSMC*s coding genes in LUAD tissues was observed.


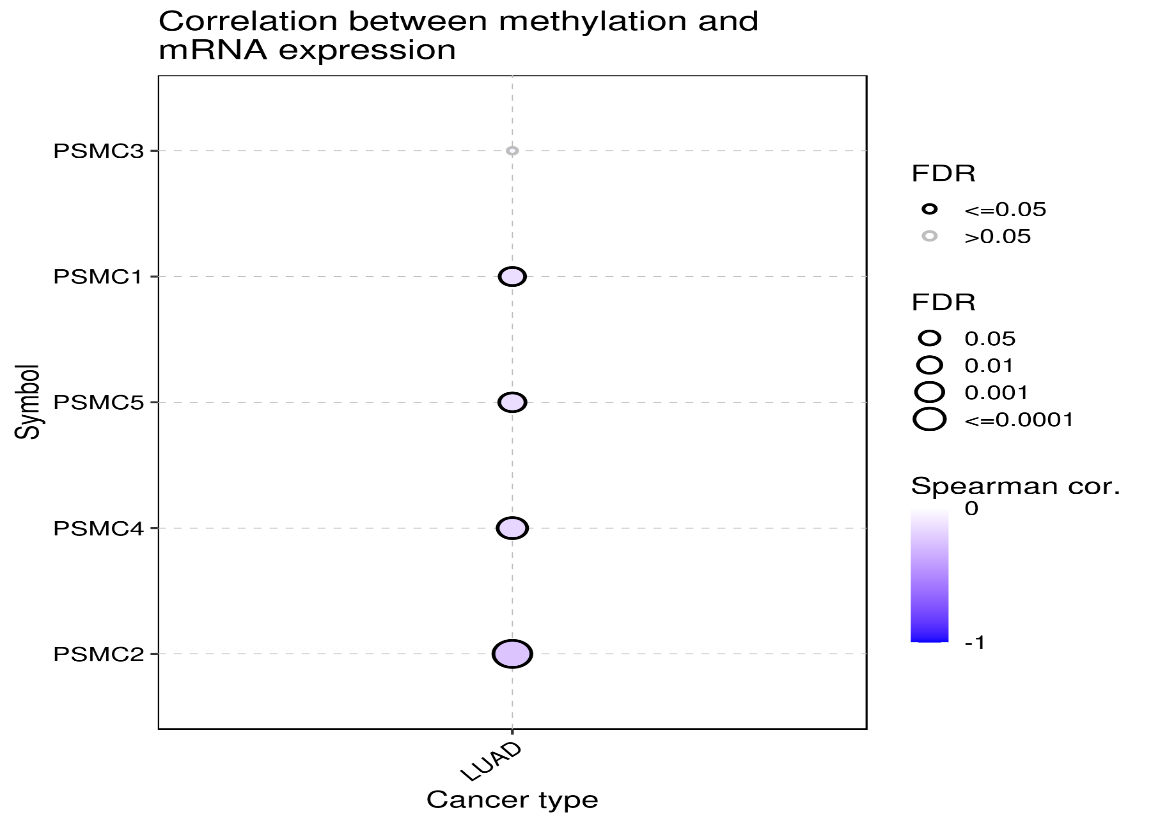


**Supplementary Figure S2:** The association between *PSMCs* methylation and mRNA expression in LUAD patients. Methylation level is negatively correlated with the mRNA expression level of *PSMCs*.

**
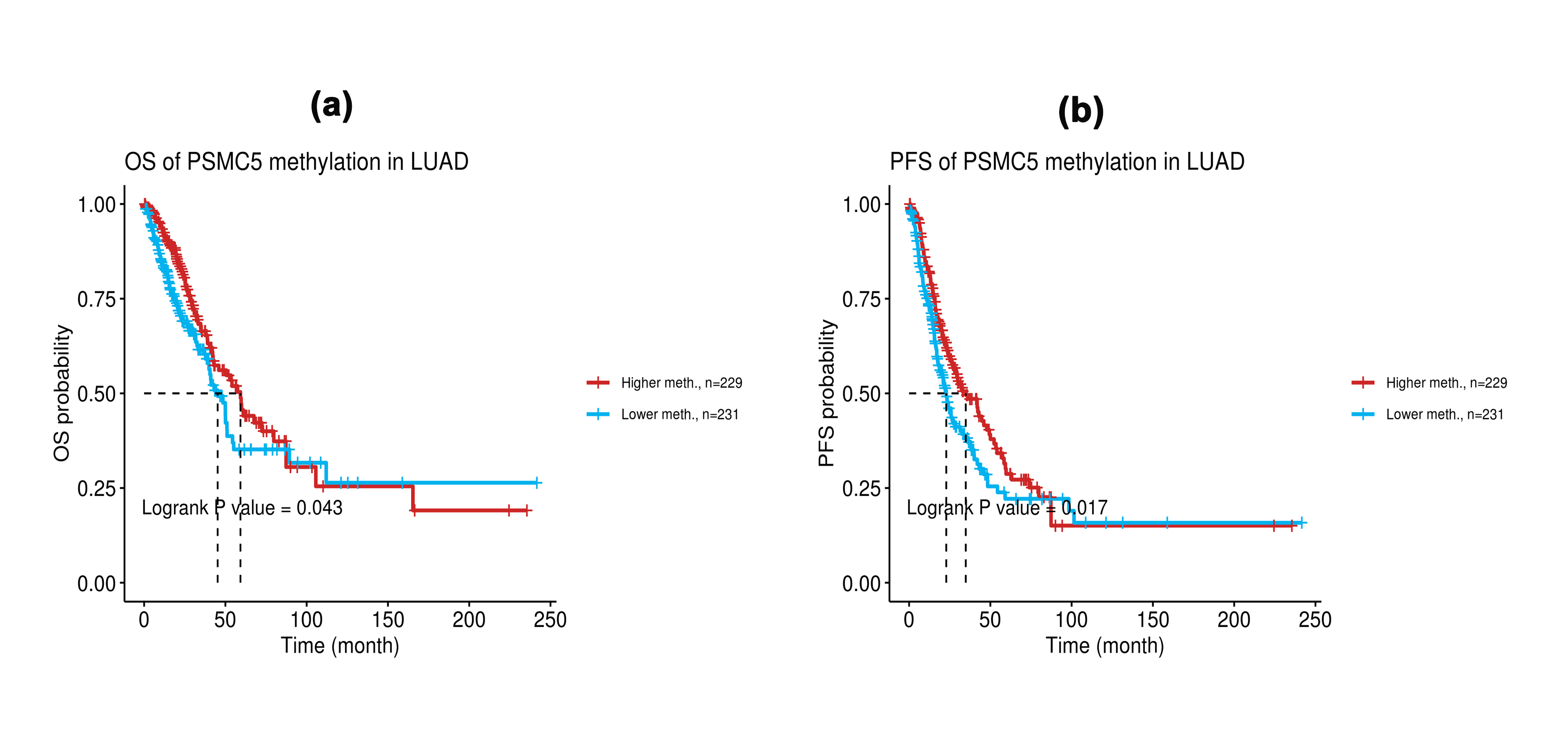
**

**Supplementary Figure S3:** The association between *PSMC*s methylation and LUAD patients’ OS and PFS. Lower methylation level of *PSMC5* was negatively correlated to the OS and PFS of LUAD patients (p<0.05).


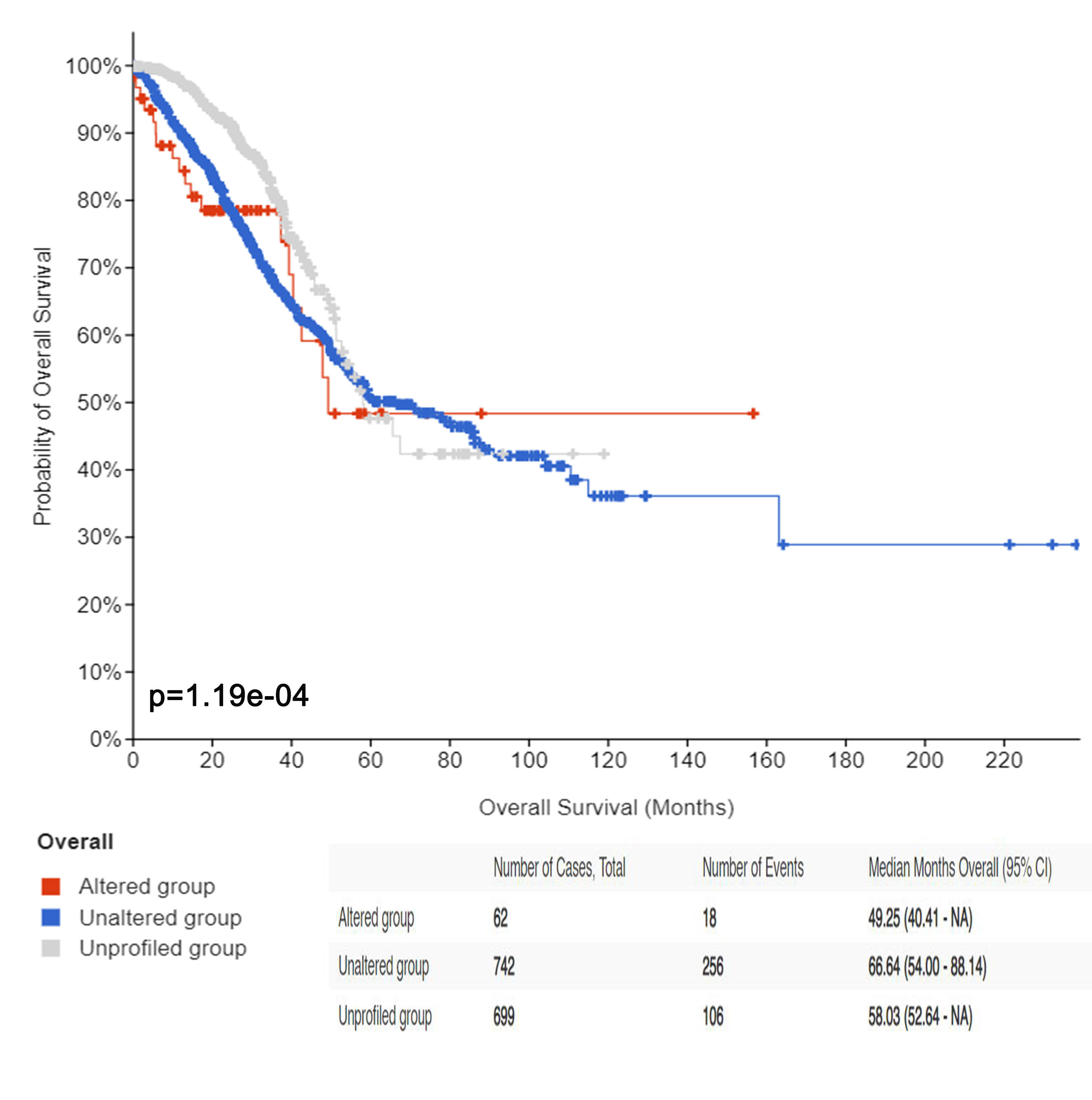


**Supplementary Figure S4:** Kaplan-Meier plot representation of the impact of *PSMC*s mutations on the OS of LUAD patients. *PSMC*s mutations were negativel correlated to the OS of LUAD patients (log rank t-test p-val: 1.19e-04).


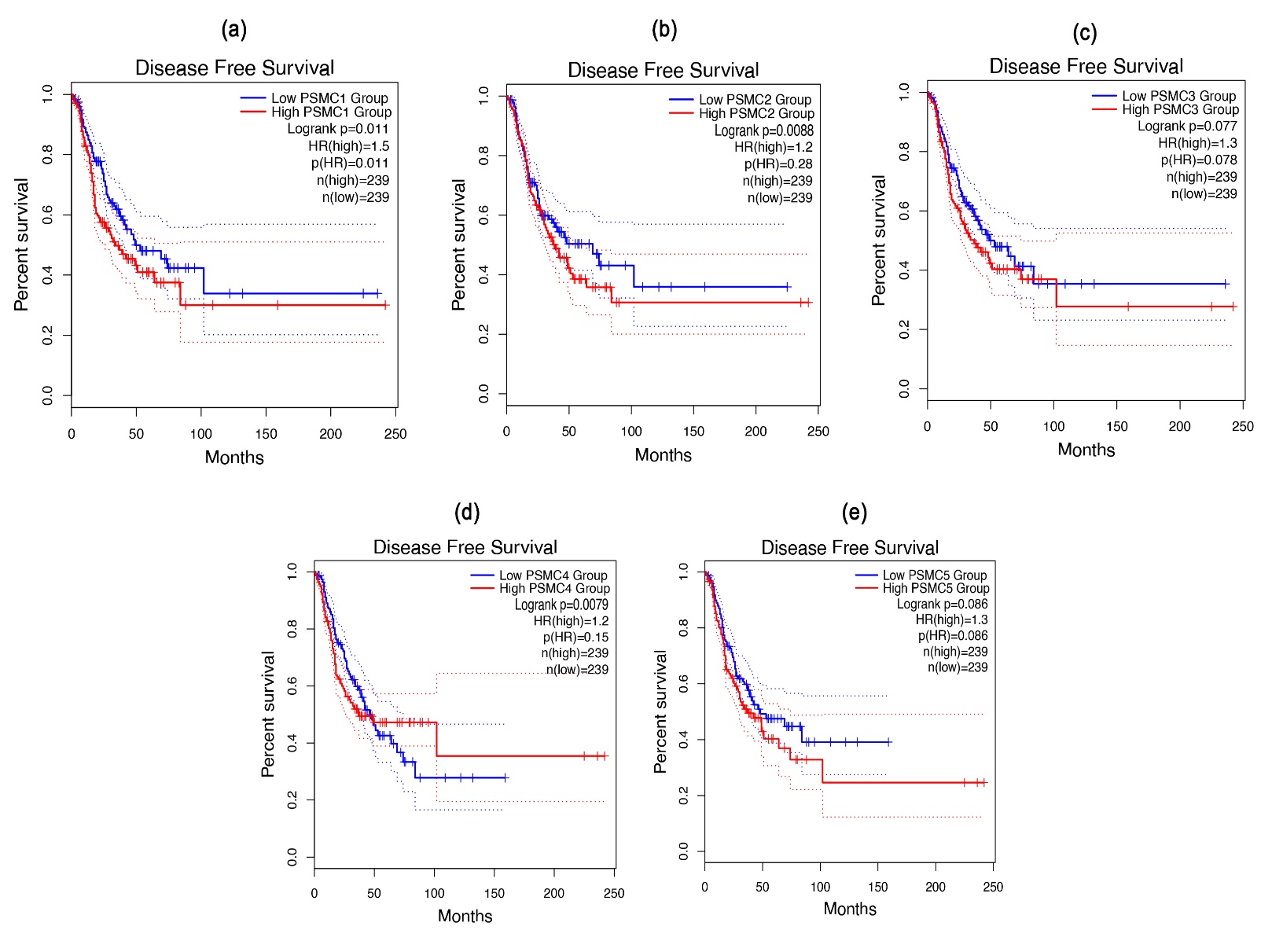


**Supplementary Figure S5:** The Kaplan-Meier plot representation of *PSMC1* (a), *PSMC2* (b), *PSMC3* (c), *PSMC4* (d) and *PSMC5* (e) expression and their relation with the RFS of LUAD patients. Significant negative association was observed between multiple *PSMC* (*PSMC1*, *PSMC2*, *PSMC4*) expression and LUAD patients’ OS (p<0.05, HR: >1.2). Red color plot represents the high *PSMC* expressing LUAD patients and the blue color plot represents the low *PSMC* expressing group. The vertical tick mark within the plot indicates an event (death).


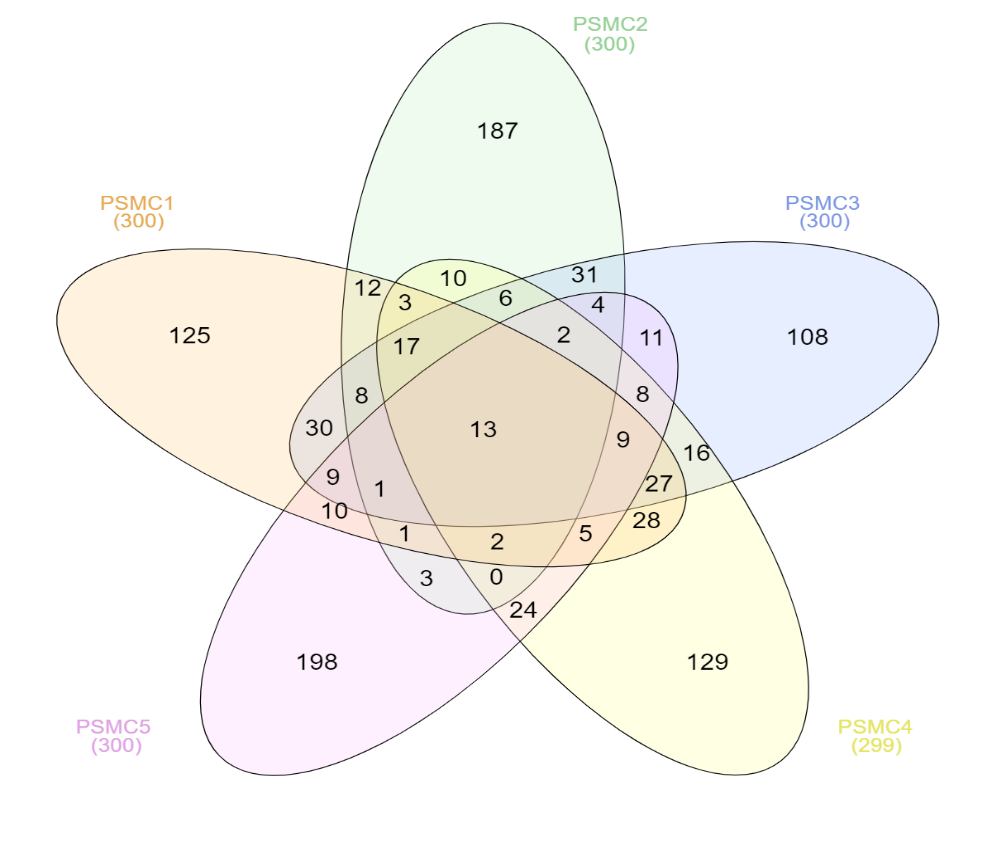


**Supplementary Figure S6:** Venn diagram representing the number of overlapping neighbor genes of *PSMC*s from top 300 selected co-expressed genes in LUAD tissues.


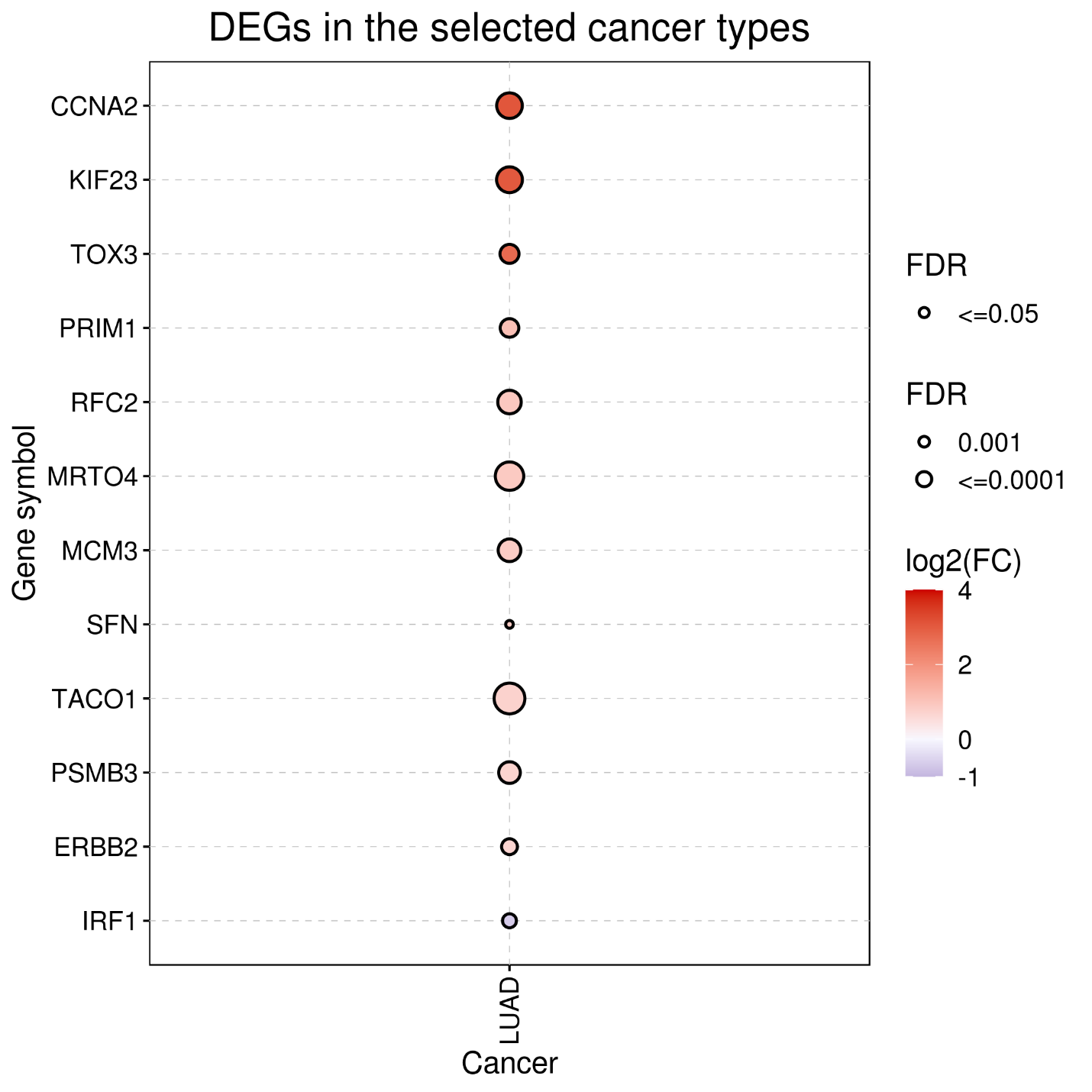


**Supplementary Figure S7:** The differential expression pattern of the overlapping neighbor genes of *PSMC* in LUAD tissues from GSCA server. All the genes showed significant overexpression in LUAD tissues compared to the normal except *IRF1* which showed under-expression. The differential expression pattern of *PDCD45* in LUAD was not found.


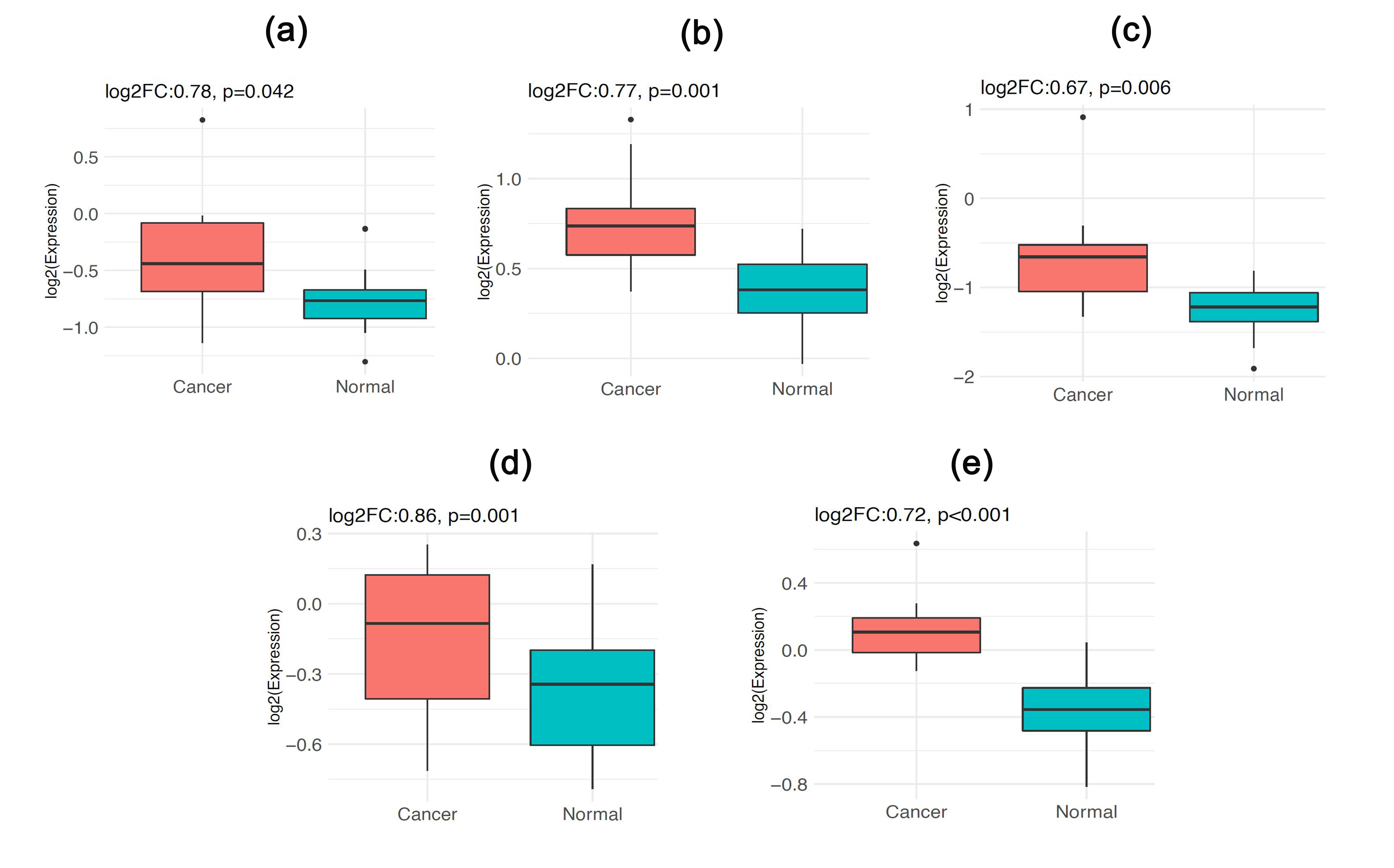


**Supplementary Figure S8:** The expression pattern of the *PSMC* genes in LUAD tissues (n=12) and adjacent normal lung tissues (n=19) obtained from GSE1037 dataset: (a) *PSMC1*, (b) *PSMC2*, (c) *PSMC3*, (d) *PSMC4*, (e) *PSMC5*. All the *PSMC*s were found to be significantly overexpressed in LUAD tissues compared to the adjacent normal lung tissues (log2FC: >0.66, p<0.05)


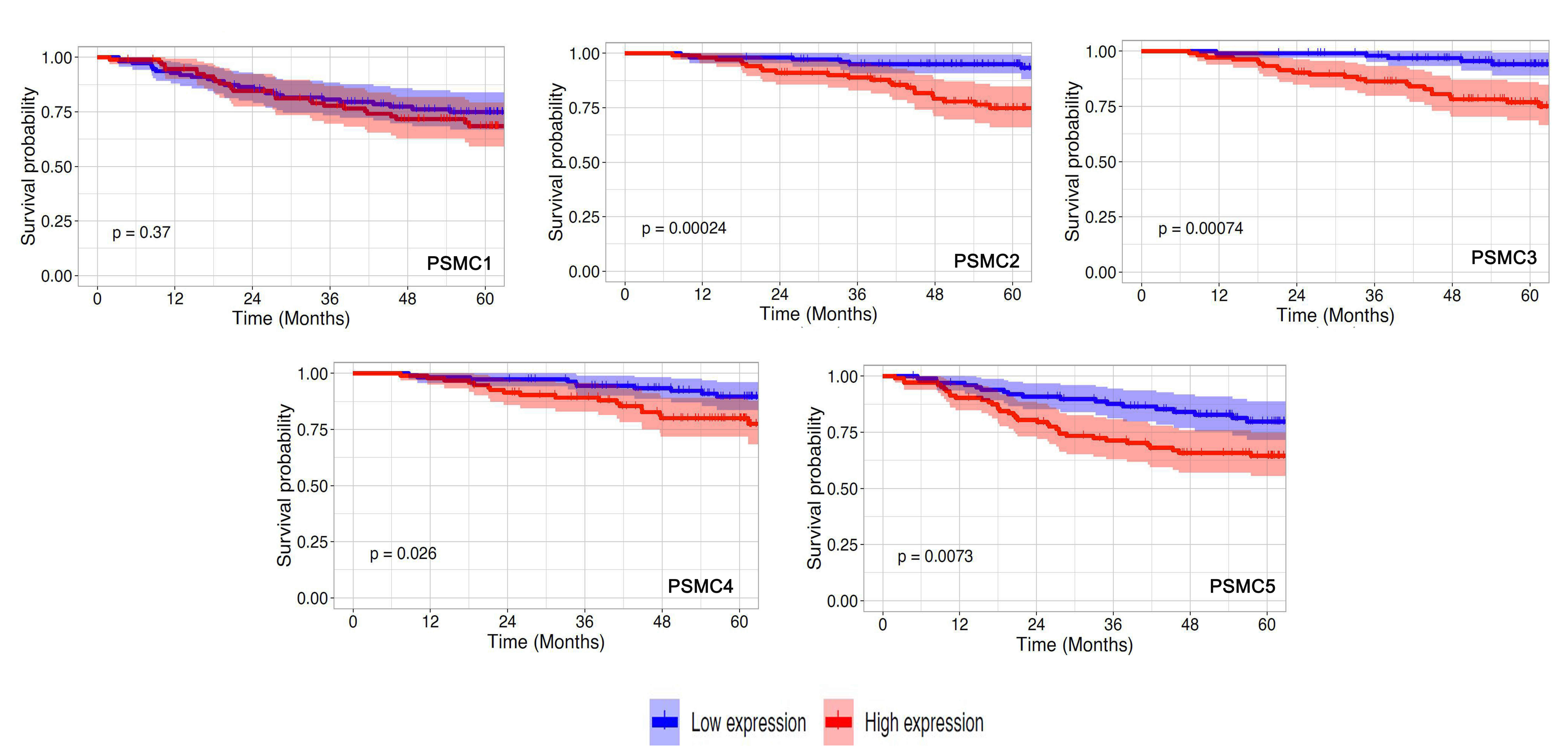


**Supplementary Figure S9:** The association between *PSMC*s expression and LUAD patients’ OS established from GSE31210 dataset. *PSMC2*-5 expression was found to have significant negative impact on the OS of LUAD patients (p<0.05).

| **Sl. No.** | **Features** | **Comparison** | **p-value** | | | | |
| --- | --- | --- | --- | --- | --- | --- | --- |
|  |  |  | ***PSMC1*** | ***PSMC2*** | ***PSMC3*** | ***PSMC4*** | ***PSMC5*** |
| 1. | **Age** | Normal-vs-Age(21-40Yrs) | **8.64E-02** | 3.21E-02 | **1.38E-01** | **1.45E-01** | 6.18E-04 |
|  |  | Normal-vs-Age(41-60Yrs) | 1.62E-12 | <1E-12 | <1E-12 | <1E-12 | <1E-12 |
|  |  | Normal-vs-Age(61-80Yrs) | 3.08E-10 | <1E-12 | <1E-12 | 1.62E-12 | <1E-12 |
|  |  | Normal-vs-Age(81-100Yrs) | 2.97E-04 | 5.64E-05 | 3.58E-03 | 1.29E-03 | 2.08E-05 |
| 2. | **Cancer Stages** | Normal-vs-Stage 1 | 1.62E-12 | <1E-12 | <1E-12 | <1E-12 | 1.62E-12 |
|  |  | Normal-vs-Stage 2 | 2.03E-08 | 3.04E-14 | 1.91E-12 | 1.62E-12 | 1.11E-16 |
|  |  | Normal-vs-Stage 3 | 7.71E-03 | 8.42E-11 | 3.17E-12 | <1E-12 | 1.24E-14 |
|  |  | Normal-vs-Stage 4 | 1.30E-03 | 1.73E-05 | 8.26E-03 | 3.54E-05 | 1.38E-04 |
| 3. | **Nodal Metastasis Status** | Normal-vs-N0 | 1.62E-12 | 1.62E-12 | 1.62E-12 | <1E-12 | 1.62E-12 |
|  |  | Normal-vs-N1 | 4.26E-07 | 1.62E-12 | 1.80E-12 | 1.62E-12 | 9.99E-16 |
|  |  | Normal-vs-N2 | 1.37E-02 | 2.96E-10 | 3.71E-10 | <1E-12 | 6.22E-12 |
|  |  | Normal-vs-N3 | **5.85E-01** | **3.35E-01** | 2.51E-06 | **1.15E-01** | 1.08E-07 |

**Supplementary Table S1:** The summary of the analysis revealing the association of *PSMCs* overexpression in relation to LUAD patients’ age, individual cancer stages and nodal metastasis status.

| **Gene Symbol** | **Immune Cell** | **Correlation Coefficient** | **P Value** | **FDR** |
| --- | --- | --- | --- | --- |
| ***PSMC1*** | B Cell | 0.099594 | 0.016802 | 0.03864 |
|  | CD4+ T Cell | -0.27175 | 3.29E-11 | 1.57E-10 |
|  | CD8+ T Cell | 0.137747 | 0.000918 | 0.003636 |
|  | DC | 0.079554 | 0.056368 | 0.111684 |
|  | Macrophage | -0.06857 | 0.100148 | 0.15146 |
|  | Monocyte | -0.01491 | 0.72105 | 0.816913 |
|  | NK | -0.07867 | 0.059176 | 0.091886 |
|  | Neutrophil | -0.04502 | 0.280715 | 0.420926 |
| ***PSMC2*** | B Cell | 0.164948 | 6.97E-05 | 0.000373 |
|  | CD4+ T Cell | -0.36278 | 2.34E-19 | 2.81E-18 |
|  | CD8+ T Cell | 0.094793 | 0.022893 | 0.054836 |
|  | DC | 0.218829 | 1.13E-07 | 1.3E-06 |
|  | Macrophage | 0.115703 | 0.005433 | 0.011418 |
|  | Monocyte | 0.062665 | 0.133054 | 0.242921 |
|  | NK | -0.14255 | 0.000601 | 0.001427 |
|  | Neutrophil | -0.12668 | 0.002318 | 0.00916 |
| ***PSMC3*** | B Cell | 0.146267 | 0.000429 | 0.001732 |
|  | CD4+ T Cell | -0.28058 | 7.01E-12 | 3.59E-11 |
|  | CD8+ T Cell | 0.152128 | 0.000248 | 0.001196 |
|  | DC | 0.12272 | 0.003177 | 0.010053 |
|  | Macrophage | -0.0156 | 0.708674 | 0.768672 |
|  | Monocyte | 0.035816 | 0.390894 | 0.53657 |
|  | NK | -0.13618 | 0.001051 | 0.00239 |
|  | Neutrophil | -0.08506 | 0.041277 | 0.097543 |
| ***PSMC4*** | B Cell | 0.216368 | 1.57E-07 | 2.09E-06 |
|  | CD4+ T Cell | -0.31289 | 1.51E-14 | 1.04E-13 |
|  | CD8+ T Cell | 0.208997 | 4.17E-07 | 4.64E-06 |
|  | DC | -0.03582 | 0.390802 | 0.516956 |
|  | Macrophage | -0.15234 | 0.000243 | 0.000678 |
|  | Monocyte | 0.095816 | 0.021455 | 0.057285 |
|  | NK | -0.16906 | 4.54E-05 | 0.000131 |
|  | Neutrophil | -0.11504 | 0.005708 | 0.019589 |
| ***PSMC5*** | B Cell | 0.125436 | 0.002563 | 0.007888 |
|  | CD4+ T Cell | -0.11052 | 0.007937 | 0.013264 |
|  | CD8+ T Cell | 0.081623 | 0.050233 | 0.104451 |
|  | DC | 0.030064 | 0.471442 | 0.594093 |
|  | Macrophage | -0.16563 | 6.49E-05 | 0.000203 |
|  | Monocyte | 0.113123 | 0.006572 | 0.021712 |
|  | NK | -0.16767 | 5.25E-05 | 0.00015 |
|  | Neutrophil | -0.0384 | 0.357639 | 0.501524 |

**Supplementary Table S2:** Summary of the association between *PSMC* mRNA expression different immune cell infiltration levels in LUAD tissues.
